# Evidence that S6K1, but not 4E-BP1, mediates skeletal muscle pathology associated with loss of A-type lamins

**DOI:** 10.1038/celldisc.2017.39

**Published:** 2017-10-31

**Authors:** Chen-Yu Liao, Sydney S Anderson, Nicole H Chicoine, Jarrott R Mayfield, Brittany J Garrett, Charlotte S Kwok, Emmeline C Academia, Yueh-Mei Hsu, Delana M Miller, Amanda M Bair, Joy A Wilson, Gabriella Tannady, Erin M Stewart, Stuart S Adamson, Junying Wang, Dominic J Withers, Brian K Kennedy

**Affiliations:** 1 Buck Institute for Research on Aging, Novato, CA, USA; 2 MRC London Institute of Medical Sciences, London, UK; 3 Institute of Clinical Sciences, Faculty of Medicine, Imperial College London, London, UK; 4 Departments of Biochemistry and Physiology, Yong Loo Lin School of Medicine, National University of Singapore, Singapore, Singapore

**Keywords:** *Lmna*^*−/−*^ mice, rapamycin, mTORC1, S6K1, 4E-BP1, lifespan, muscle

## Abstract

The mechanistic target of rapamycin (mTOR) signaling pathway plays a central role in aging and a number of different disease states. Rapamycin, which suppresses activity of the mTOR complex 1 (mTORC1), shows preclinical (and sometimes clinical) efficacy in a number of disease models. Among these are *Lmna*^*−/−*^ mice, which serve as a mouse model for dystrophy-associated laminopathies. To confirm that elevated mTORC1 signaling is responsible for the pathology manifested in *Lmna*^*−/−*^ mice and to decipher downstream genetic mechanisms underlying the benefits of rapamycin, we tested in *Lmna*^*−/−*^ mice whether survival could be extended and disease pathology suppressed either by reduced levels of S6K1 or enhanced levels of 4E-BP1, two canonical mTORC1 substrates. Global heterozygosity for *S6K1* ubiquitously extended lifespan of *Lmna*^*−/−*^ mice (*Lmna*^*−/−*^ *S6K1*^*+/−*^ mice). This life extension is due to improving muscle, but not heart or adipose, function, consistent with the observation that genetic ablation of *S6K1* specifically in muscle tissue also extended survival of *Lmna*^*−/−*^ mice. In contrast, whole-body overexpression of 4E-BP1 shortened the survival of *Lmna*^*−/−*^ mice, likely by accelerating lipolysis. Thus, rapamycin-mediated lifespan extension in *Lmna*^*−/−*^ mice is in part due to the improvement of skeletal muscle function and can be phenocopied by reduced S6K1 activity, but not 4E-BP1 activation.

## Introduction

The mechanistic target of rapamycin (mTOR) kinase is a central regulator of growth factor signaling and metabolism [1–3] and is closely linked to aging and a wide range of diseases [4]. The mTOR protein is a component of two complexes, the mTOR complex 1 (mTORC1) and complex 2 (mTORC2). The best-defined substrates of the mTORC1 complex are S6K1 (ribosomal protein S6 protein kinase 1) and 4E-BP1 (eIF4E (eukaryotic translation initiation factor 4E)-binding protein 1) [5], both of which are important in the control of translation initiation [6]. Activation of the mTORC1 signaling cascade results in the phosphorylation of downstream substrates such as S6K1 and 4E-BP1, which in turn affect protein synthesis. Specifically, phosphorylation of S6K1 results in its activation and the subsequent phosphorylation of ribosomal protein S6 (rpS6), as well as other components of the translation machinery, whereas phosphorylation of 4E-BP1 disrupts its binding to eIF4E, freeing this initiation factor to promote cap-dependent translation [7].

Mutations in A-type lamins are associated with a range of dystrophic and progeroid syndromes in humans [8], including dilated cardiomyopathy with conduction-system disease (CMD1A) [9], Emery-Dreifuss muscular dystrophy (EDMD2/3) [10], familial partial lipodystrophy [11]. and Hutchinson-Gilford progeria syndrome [12]. *Lmna*^*−/−*^ mice were generated nearly two decades ago to better understand the role of A-type lamins in nuclear organization and disease [13]. Despite a more recent report that these mice express a modified *LMNA* transcript and are actually hypomorphs [14], these mice have served as a workhorse disease model for the dystrophic syndromes. *Lmna*^*−/−*^ mice rapidly develop dilated cardiomyopathy and muscular dystrophy, resulting in death by 6–8 weeks [13]. Recently, we demonstrated that rapamycin reverses elevated mTORC1 signaling in multiple tissues and rescues pathogenesis of dilated cardiomyopathy, skeletal muscle dystrophy and lipodystrophy, and as a result doubles the survival of *Lmna*^*−/−*^ mice [15, 16]. Given the multiple downstream substrates of mTORC1, it is critical to identify those that have a role in *Lmna*-mediated pathogenesis. Furthermore, understanding about how rapamycin suppresses mTORC1 and rescues pathologies in *Lmna*^*−/−*^ mice may lead to better strategies to target the mTORC1 pathway in other disease states, as well as aging itself.

Here, we assess the role of two key mTORC1 substrates, S6K1 and 4E-BP1, in *Lmna*^*−/−*^ mice, finding that S6K1 is the more important mediator of pathogenesis and early mortality. Moreover, our findings indicate that a reduction in S6K1 activity in muscle underlies the benefits to these mice, indicating that altered skeletal muscle function is a contributor to mortality in the *Lmna*^*−/−*^ mouse model of laminopathies.

## Results

### Whole-body knockdown of S6K1 extends survival of *Lmna*^*−/−*^ mice

**Figure 1:**
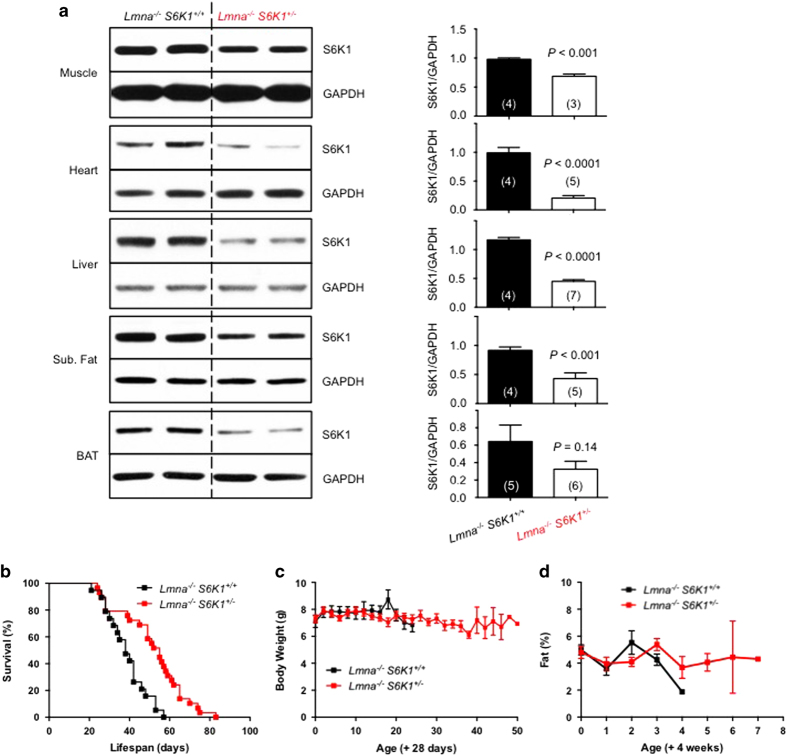
Whole-body genetic knockdown of *S6K1* extends survival of *Lmna*^*−/−*^ mice. (**a**) Western blots of S6K1 protein expression in muscle (gastrocnemius), heart, liver, subcutaneous (Sub.) fat and brown adipose tissue (BAT). Representative blot derived from two mice for each genotype. Glyceraldehyde 3-phosphate dehydrogenase (GAPDH) was used as loading control. Relative S6K1 protein levels (normalized to GAPDH) were quantified. Each value is mean±s.e.m. for the number of mice indicated in parentheses*. P-*values were derived from unpaired two-tailed Student’s *t*-test. (**b**) Kaplan–Meier survival plot of *Lmna*^*−/−*^ *S6K1*^*+/+*^ (*n*=19, black) and *Lmna*^*−/−*^ *S6K1*^*+/−*^ (*n*=29, red) mice. Data from males and females are combined. Symbols represent individual mice. Survival of *Lmna*^*−/−*^ *S6K1*^*+/−*^ mice is significantly longer than *Lmna*^*−/−*^ *S6K1*^*+/+*^ mice (*P*<0.0001 by log-rank test), resulting in a 33% increase in mean lifespan (51.5 vs 38.6 days). (**c**) Body weight (BW) of *Lmna*^*−/−*^ *S6K1*^*+/+*^ (started with *n*=12, black) and *Lmna*^*−/−*^ *S6K1*^*+/−*^ (started with *n*=19, red) mice were measured every other day from 4 weeks of age. (**d**) Adiposity (percent body fat) was measured weekly ((fat mass/BW*)*×100) from *Lmna*^*−/−*^ *S6K1*^*+/+*^ (started with *n*=8, black) and *Lmna*^*−/−*^ *S6K1*^*+/−*^ (started with *n*=19, red) mice. Symbols represent mean BW or percent body fat±s.e.m.

Previously, we reported that elevated mTORC1 signaling is responsible for many of the pathologies manifested in *Lmna*^*−/−*^ mice, and that rapamycin, which suppresses mTORC1 signaling, extends survival by rescuing those phenotypes [15, 16]. In the first step to identify the downstream pathways/targets that mediate life extension by rapamycin in *Lmna*^*−/−*^ mice, we tested the hypothesis that genetic ablation of *S6K1* may be protective in the *Lmna*^*−/−*^ mice, phenocopying the benefits of rapamycin. We crossed *Lmna*^*+/−*^ mice with *S6K1*^*+/−*^ mice [17], generating both *Lmna*^*−/−*^ *S6K1*^*+/−*^ and *Lmna*^*−/−*^ *S6K1*^*−/−*^ double-mutant mice and assessed their phenotypes (Supplementary Figure S1A). Western blot analysis of a broad spectrum of tissues in *Lmna*^*−/−*^ *S6K1*^*+/−*^ and *Lmna*^*−/−*^ *S6K1*^*−/−*^ (not shown) indicated that S6K1 protein level is reduced or absent (respectively) in disease-linked tissues of *Lmna*^*−/−*^ mice: skeletal muscle, heart, liver, subcutaneous fat (white adipose tissue, WAT) and brown adipose tissue (BAT) (Figure 1a).

First, we compared the survival of *Lmna*^*−/−*^ with 0, 1 or 2 copies of *S6K1*. Interestingly, complete deletion of *S6K1* did not enhance the survival of *Lmna*^*−/−*^ mice (Supplementary Figure S2G; see below), whereas *Lmna*^*−/−*^ *S6K1*^*+/−*^ mice lived significantly longer than *Lmna*^*−/−*^ control mice (33% extension of mean lifespan, *P*<0.0001 by log-rank test) (Figure 1b), and this result was statistically significant in both sexes (Supplementary Figure S2A, D). Therefore, *Lmna*^*−/−*^ mice heterozygous for *S6K1*, a major downstream substrate of mTORC1 signaling, partially phenocopy the life extension in *Lmna*^*−/−*^ mice by rapamycin, which doubled the survival of *Lmna*^*−/−*^ mice [16].

Previously, we observed that rapamycin treatment led to improved maintenance of adiposity in *Lmna*^*−/−*^ mice, underscoring its critical role in their survival [16]. Specifically, enhanced lipolysis in WAT and deficient thermogenesis in BAT are at least partially rescued by rapamycin in *Lmna*^*−/−*^ mice. Unlike rapamycin, however, the body weight (BW) and fat content of long-lived *Lmna*^*−/−*^ *S6K1*^*+/−*^ mice are indistinguishable from *Lmna*^*−/−*^ littermate controls (Figure 1c and d, Supplementary Figure S2B, C, E and F). These results are further supported by the observation that the lipid metabolism of long-lived *Lmna*^*−/−*^ *S6K1*^*+/−*^ mice is not changed. For instance, we reported that levels of adipose triglyceride lipase (ATGL) are elevated in WAT of *Lmna*^*−/−*^ mice, and here show that they are unaltered by *S6K1* heterozygosity (Supplementary Figure S3A). Similarly, the low levels of uncoupling protein 1 (UCP1) in BAT are not rescued (Supplementary Figure S3B). This is in spite of the fact that phosphorylation of ribosomal protein S6 (rpS6) protein (p-S6 S240/244), a well-documented readout of mTORC1 and S6K1 activity, is suppressed in both WAT and BAT (although not statistically significant) in *Lmna*^*−/−*^ *S6K1*^*+/−*^ mice (Supplementary Figure S3A, B). Thus, reduced S6K1 signaling in adipose tissues does not likely underlie the enhanced survival of *Lmna*^*−/−*^ *S6K1*^*+/−*^ mice.

Analysis of BW and composition revealed that double knockout mice (*Lmna*^*−/−*^ *S6K1*^*−/−*^) were consistently smaller than age-matched *Lmna*^*−/−*^ littermates (Supplementary Figure S2H and I). This smaller body size, which is analogous to the phenotypes of *S6K1*^*−/−*^ mice compared to littermate controls [18], may exacerbate dystrophic phenotypes of *Lmna*^*−/−*^ mice and thus override the potentially beneficial effects derived from reduced S6K1 signaling (see Discussion). Given a lower-than-expected number of double knockout mice obtained from crosses (Supplementary Figure S1A) and the unaltered lifespan (Supplementary Figure S2G), we did not further characterize the *Lmna*^*−/−*^ *S6K1*^*−/−*^ mice, focusing instead on their long-lived *Lmna*^*−/−*^ *S6K1*^*+/−*^ counterparts.

Rapamycin induces glucose intolerance as indicated by glucose tolerance test (GTT) in wild-type mice (WT; *Lmna*^*+/+*^ *S6K1*^*+/+*^) [19] (Supplementary Figure S4A). In contrast, *Lmna*^*−/−*^ mice are hypoglycemic [20] and have increased sensitivity upon glucose infusion (Supplementary Figure S4A). After 1 week of rapamycin treatment, *Lmna*^*−/−*^ mice had reduced glucose tolerance compared with untreated controls; however, they were comparable to untreated *Lmna*^*+/+*^ mice (Supplementary Figure S4A). After 3 weeks of treatment, rapamycin induces glucose intolerance in *Lmna*^*−/−*^ mice (Supplementary Figure S4A). Given the lack of response to glucose challenge in *Lmna*^*−/−*^ mice, it is possible that the glucose intolerance and hyperglycemia induced by rapamycin may play a positive role in life extension in *Lmna*^*−/−*^ mice. *S6K1*^*−/−*^ mice are long-lived, and in these mice glucose tolerance is improved in 600-day-old females but impaired in 8-week-old mice [18]. In contrast to rapamycin-treated *Lmna*^*−/−*^ mice (Supplementary Figure S4A), however, long-lived *Lmna*^*−/−*^ *S6K1*^*+/−*^ mice have comparable glucose profiles in response to GTT to *Lmna*^*−/−*^ mice (Supplementary Figure S4B). These results suggest life extension in *Lmna*^*−/−*^ *S6K1*^*+/−*^ mice is not owing to altered glucose sensitivity and support the hypothesis that, whereas rapamycin targets multiple metabolic tissues to extend lifespan in *Lmna*^*−/−*^ mice [15, 16], a reduction in S6K1 may only provide a subset of those benefits.

### Improved cardiac function is not observed in long-lived *Lmna*^*−/−*^*S6K1*^*+/−*^ mice

The primary cause of death for *Lmna*^*−/−*^ mice has been reported to be cardiac dysfunction [21], and rapamycin at least partially reverses this phenotype [15]. Given that metabolic parameters were not altered in *Lmna*^*−/−*^ *S6K1*^*+/−*^ mice, we next speculated that these mice may have improved cardiac function. S6K1 protein levels are reduced in heart tissue of *Lmna*^*−/−*^ *S6K1*^*+/−*^ mice by at least 50%, as expected (Figure 1a). Thus, we evaluated the cardiac function by transthoracic echocardiography in long-lived *Lmna*^*−/−*^ *S6K1*^*+/−*^ mice. In our experimental setting, we showed that *Lmna*^*−/−*^ mice have cardiac functional deficits, indicated by increased left ventricular (LV) end-systolic diameter and LV end-diastolic diameter, compared with wild-type mice (WT; *Lmna*^*+/+*^ *S6K1*^*+/+*^) at 5~6 weeks of age (Supplementary Figure S5A) [15]. However, improved cardiac function was not observed in long-lived *Lmna*^*−/−*^ *S6K1*^*+/−*^ mice. All the parameters, including LV end-systolic diameter, LV end-diastolic diameter, myocardial performance index, ejection fraction, fractional shortening and cardiac output, are indistinguishable between control *Lmna*^*−/−*^ *S6K1*^*+/+*^ and long-lived *Lmna*^*−/−*^ *S6K1*^*+/−*^ mice (Supplementary Figure S5A). At the molecular level, rapamaycin decreases the amount of desmin in cardiac tissue of *Lmna*^*−/−*^ mice [15]. However, desmin was not decreased in cardiac tissue of long-lived *Lmna*^*−/−*^ *S6K1*^*+/−*^ mice (Supplementary Figure S5B). Thus, the life extension in *Lmna*^*−/−*^ *S6K1*^*+/−*^ mice is not due to detectable improvements in cardiac function.

### Improved skeletal muscle function in long-lived *Lmna*^*−/−*^*S6K1*^*+/−*^ mice

**Figure 2:**
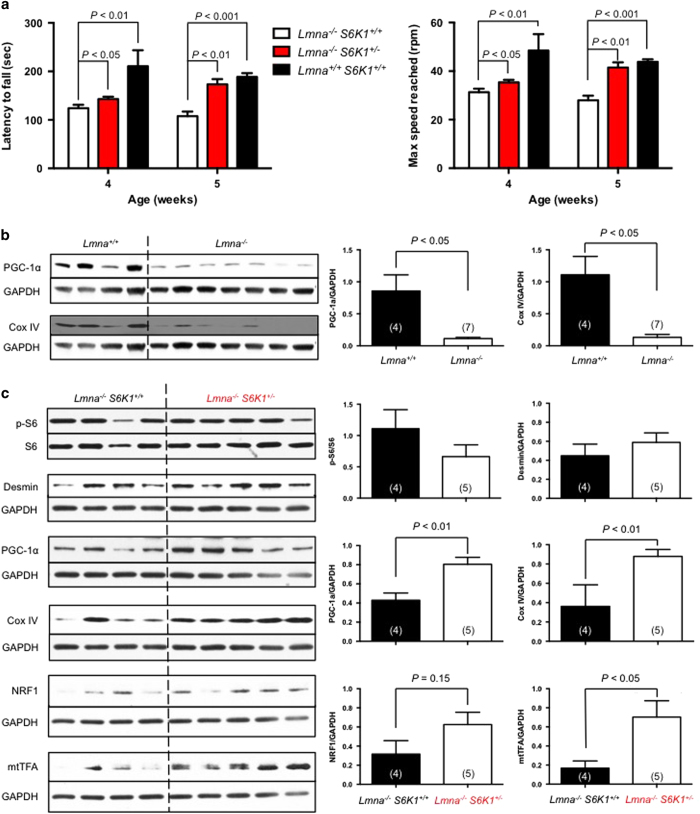
Improved muscle function in long-lived *Lmna*^*−/−*^ *S6K1*^*+/−*^ mice. (**a**) Analysis of muscle function by rotarod test. There was a significant decrease in latency to fall and maximum speed reached in *Lmna*^*−/−*^ (*Lmna*^*−/−*^ *S6K1*^*+/+*^) mice compared to wild-type (WT; *Lmna*^*+/+*^ *S6K1*^*+/+*^) mice. Long-lived *Lmna*^*−/−*^ *S6K1*^*+/−*^ mice experienced significantly increased latency to fall and maximum speed reached compared to *Lmna*^*−/−*^ mice. (**b**) PGC-1α and Cox IV protein levels in muscle (gastrocnemius) tissue of *Lmna*^*+/+*^ and *Lmna*^*−/−*^ mice. Relative PGC-1α and Cox IV protein levels (normalized to GAPDH) were quantified. (**c**) Signaling through the mTORC1 pathway, indicated by p-S6, in muscle (gastrocnemius) tissue of long-lived *Lmna*^*−/−*^ *S6K1*^*+/−*^ mice. Relative p-S6 levels (normalized to S6) and relative desmin, PGC-1α, Cox IV, NRF1 and mtTFA protein levels (normalized to GAPDH) were quantified. Each value is mean±s.e.m. for replicate numbers indicated in parentheses, and statistical significance was determined by unpaired two-tailed Student’s* t-*test.

Since partial knockdown of *S6K1* improves the survival of *Lmna*^*−/−*^ mice (Figure 1b), and neither cardiac function nor metabolic parameters are improved in long-lived *Lmna*^*−/−*^ *S6K1*^*+/−*^ mice (Supplementary Figure S4B), we further evaluated whether genetic reduction of *S6K1* rescues skeletal muscle deficits in *Lmna*^*−/−*^ mice. If elevated mTORC-S6K1 activity contributes to the muscle dystrophy and this phenotype also reduces survival, then reduced S6K1 signaling may be exerting its protective effect in skeletal muscle. Our findings are consistent with this hypothesis. For instance, muscle function was improved in long-lived *Lmna*^*−/−*^ *S6K1*^*+/−*^ mice evaluated by rotarod at 4 and 5 weeks of age with double-mutant mice displaying both enhanced latency to fall and increased maximum speed reached (Figure 2a).

Consistent with a prior report, we find reduced rotarod performance in *Lmna*^*−/−*^ mice [15]. (Figure 2a). Muscular dystrophy in *Lmna*^*−/−*^ may also be driven by reduced levels of peroxisome proliferator-activated receptor gamma coactivator-1-alpha (PGC-1α), a master regulator of mitochondrial biogenesis [22]. (Figure 2b). We interrogated *Lmna*^*−/−*^ *S6K1*^*+/−*^ mice to see if PGC-1α levels were affected by reduced S6K1 activity. Interestingly, we found PGC-1α protein levels were restored in muscle tissue (gastrocnemius) of long-lived *Lmna*^*−/−*^ *S6K1*^*+/−*^ mice (Figure 2b and c). Furthermore, mitochondrial protein subunit 4 of cytochrome c oxidase complex (Cox IV), a nuclear-encoded mitochondrial protein of the electron transport chain, is reduced in skeletal muscle of *Lmna*^*−/−*^ mice and also restored in *Lmna*^*−/−*^ *S6K1*^*+/−*^ mice (Figure 2b and c). This improved mitochondrial function in skeletal muscle of long-lived *Lmna*^*−/−*^ *S6K1*^*+/−*^ mice is further supported by a trend toward increased nuclear respiratory factor 1 (NRF1) and increased mitochondrial transcription factor A (mtTFA) (Figure 2c). NRF1 is a PGC-1α-inducible transcription activator for the gene encoding cytochrome c [23]. PGC-1α also could activate the expression of mtTFA through the coactivation of NRF1-mediated transcription [24]. mtTFA is a nuclear-encoded gene product that is imported into the mitochondrial for mitochondrial biogenesis, including the replication and transcription of mitochondrial DNA [24]. However, the amount of desmin was not reduced in skeletal muscle of long-lived *Lmna*^*−/−*^ *S6K1*^*+/−*^ mice (Figure 2c). Of note, we detect a modest trend toward reduced phosphorylation of the S6K1 substrate, ribosomal protein S6 (rpS6), although it was not statistically significant (Figure 2c). We speculate that phosphorylation is maintained by S6K2 and/or other reported kinases [25, 26]. and it is also possible that the rescue in skeletal muscle may occur through phosphorylation of other S6K1 substrates. The rescue of PGC-1α in skeletal muscle was not observed in cardiac tissue (Supplementary Figure S5B), further supporting our findings that cardiac function is not improved in long-lived *Lmna*^*−/−*^ *S6K1*^*+/−*^ mice.

### Muscle-specific S6K1 knockdown improves survival in *Lmna*^*−/−*^ mice

**Figure 3:**
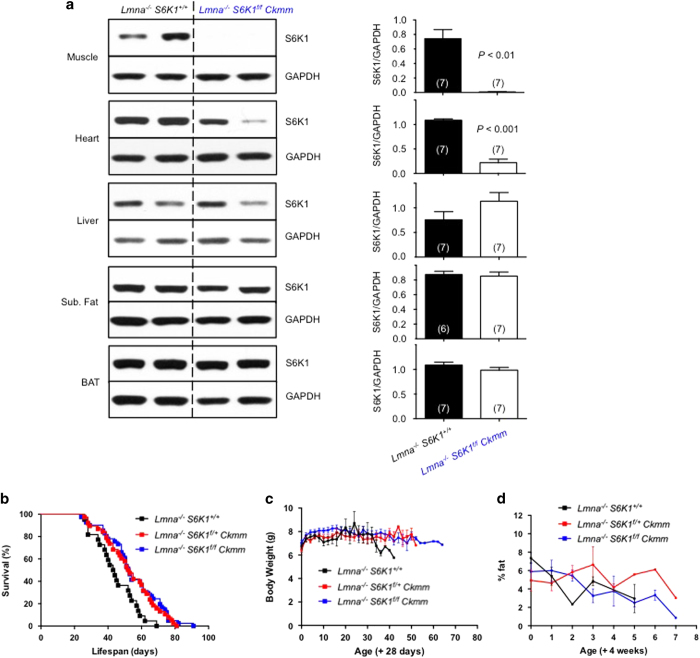
Muscle-specific *S6K1* knockout improves survival of *Lmna*^*−/−*^ mice. (**a**) Western blots of S6K1 protein expression in muscle (gastrocnemius), heart, liver, subcutaneous (Sub.) fat and brown adipose tissue (BAT). Representative blot derived from two mice for each genotype. Glyceraldehyde 3-phosphate dehydrogenase (GAPDH) was used as loading control. Relative S6K1 protein levels (normalized to GAPDH) were quantified. Each value is mean±s.e.m. for the number of mice indicated in parentheses. *P*-values were derived from unpaired two-tailed Student’s* t-*test. (**b**) Kaplan–Meier survival plot of *Lmna*^*−/−*^ *S6K1*^*+/+*^ (*n*=24, black), *Lmna*^*−/−*^ *S6K1*^*f/+*^ *Ckmm* (*n*=40, red) and *Lmna*^*−/−*^ *S6K1*^*f/f*^ *Ckmm* (*n*=42, blue) mice. Survival is significantly increased in *Lmna*^*−/−*^ *S6K1*^*f/+*^ *Ckmm* (red) and *Lmna*^*−/−*^ *S6K1*^*f/*f^ *Ckmm* (blue) mice (*P*<0.01 and *P*<0.001, respectively, by log-rank test). Data from males and females are combined. Symbols represent individual mice. (**c**) Body weight (BW) of *Lmna*^*−/−*^ *S6K1*^*+/+*^ (started with *n*=22, black), *Lmna*^*−/−*^ *S6K1*^*f/+*^ *Ckmm* (started with *n*=38, red) and *Lmna*^*−/−*^ *S6K1*^*f/f*^ *Ckmm* (started with *n*=39, blue) mice were measured from 4 weeks of age. (**d**) Adiposity (percent body fat) was measured weekly ((fat mass/BW)×100) from *Lmna*^*−/−*^ *S6K1*^*+/+*^ (started with *n*=4, black) and *Lmna*^*−/−*^ *S6K1*^*f/+*^ *Ckmm* (started with *n*=5, red) and *Lmna*^*−/−*^ *S6K1*^*f/f*^ *Ckmm* (started with *n*=8, blue) mice from 4 weeks of age.

Given our findings that *Lmna*^*−/−*^ mice with reduced S6K1 activity have enhanced survival and improved skeletal muscle (but not cardiac and metabolic) function, we decided to target S6K1 specifically in skeletal muscle to provide a more direct test of the role of elevated mTORC1 signaling in this tissue. Thus, we generated a muscle-specific *S6K1* knockout mice in the *Lmna*^*−/−*^ context to test this hypothesis (Supplementary Figure S1B). A breeding strategy utilizing *Lmna*^*+/−*^ mice [13], mice bearing one allele floxed *S6K1* gene (*S6K1*^*flox/+*^ or *S6K1*^*f/+*^ mice) [27], mice expressing Cre recombinase under the control of the muscle creatine kinase promoter (*Ckmm-Cre*) [28] was utilized to generate muscle-specific *S6K1* knockdown in *Lmna*^*−/−*^ background (that is, *Lmna*^*−/−*^ *S6K1*^*flox/flox*^ *Ckmm* or *Lmna*^*−/−*^ *S6K1*^*f/f*^ *Ckmm* mice) (Supplementary Figure S1B). As expected, western blot analysis of a broad spectrum of tissues showed that the S6K1 protein level is reduced in skeletal muscle and to some extent in heart, but not in liver, WAT or BAT of *Lmna*^*−/−*^ *S6K1*^*f/f*^ *Ckmm* mice (Figure 3a).

Interestingly and consistent with our hypothesis, both *Lmna*^*−/−*^ *S6K1*^*f/+*^ *Ckmm* and *Lmna*^*−/−*^ *S6K1*^*f/f*^ *Ckmm* mice outlived control *Lmna*^*−/−*^ mice (Figure 3b). This survival study indicates that *Lmna*^*−/−*^ *S6K1*^*f/+*^ *Ckmm* and *Lmna*^*−/−*^ *S6K1*^*f/f*^ *Ckmm* mice resemble the conventional deletion of one copy of *S6K1* in *Lmna*^*−/−*^ mice (*Lmna*^*−/−*^ *S6K*^*+/−*^ mice) (Figure 1b). Of note, the *S6K1* gene with the floxed allele alone or the presence of the *Ckmm* gene alone could in theory have been a confounding factor that contributed to longer lifespan of *Lmna*^*−/−*^ *S6K1*^*f/+*^ *Ckmm* and *Lmna*^*−/−*^ *S6K1*^*f/f*^ *Ckmm* mice. However, the lifespan of the following mice derived from our breeding strategy is indistinguishable from *Lmna*^*−/−*^ mice: *Lmna*^*−/−*^ *S6K1*^*f/+*^, *Lmna*^*−/−*^ *S6K1*^*f/f*^ and *Lmna*^*−/−*^ *S6K1*^*+/+*^ *Ckmm* have identical survival curves (Supplementary Figure S6). Given *Lmna*^*−/−*^ *S6K1*^*f/f*^ *Ckmm* mice lived longest in terms of mean lifespan (although no statistically different from *Lmna*^*−/−*^ *S6K1*^*f/+*^ *Ckmm* mice; Supplementary Figure S6), we compared *Lmna*^*−/−*^ *S6K1*^*+/+*^ with *Lmna*^*−/−*^ *S6K1*^*f/f*^ *Ckmm* mice (both were derived from *Lmna*^*−/−*^ *S6K1*^*f/+*^×*Lmna*^*−/−*^ *S6K1*^*f/+*^ *Ckmm* crossing; Supplementary Figure S1B) for the remainder of the study.

As with the *Lmna*^*−/−*^ *S6K1*^*+/−*^ mice and unlike rapamycin-treated *Lmna*^*−/−*^ mice [16], BW and fat content of long-lived *Lmna*^*−/−*^ *S6K1*^*f/f*^ *Ckmm* mice was comparable to *Lmna*^*−/−*^ mice (Figure 3c and d). Previously, we showed that elevated lipolysis in WAT, indicated by higher levels of ATGL, may underlie the lipodystrophic phenotype in *Lmna*^*−/−*^ mice [16]. Interestingly, ATGL is further elevated in long-lived *S6K1*^*f/f*^ *Ckmm* mice (Supplementary Figure S7A), whereas thermogenic protein UCP1 is indistinguishable in BAT (Supplementary Figure S7B). The reasons for this elevated lipolysis are unclear given that we did not observe a dramatic change in BW and adiposity (Figure 3c and d). Nonetheless, consistent with the data from long-lived *Lmna*^*−/−*^ *S6K1*^*+/−*^ mice (Figure 1c and d), the loss of adipose tissue is not rescued in the *Lmna*^*−/−*^ *S6K1*^*f/f*^ *Ckmm* mice.

### Muscle function is improved in *Lmna*^*−/−*^ mice with muscle-specific S6K1 knockout

**Figure 4:**
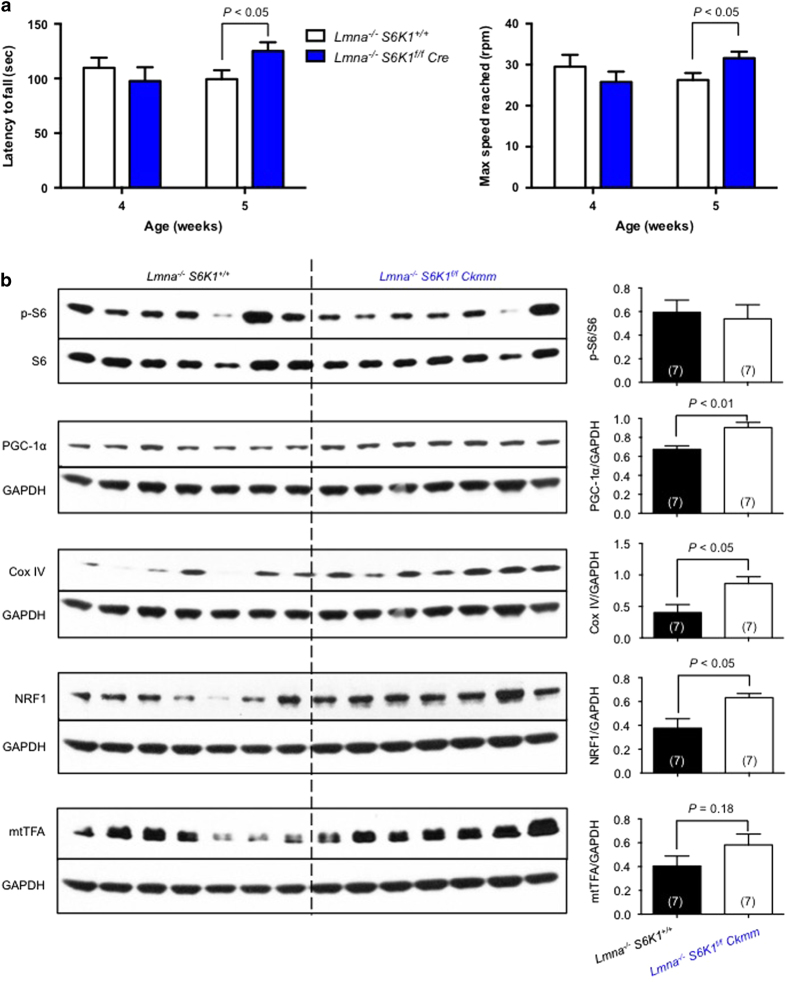
Improved muscle function in long-lived *Lmna*^*−/−*^ *S6K1*^*f/f*^ *Ckmm* mice. (**a**) Analysis of muscle function by rotarod test. Long-lived *Lmna*^*−/−*^ *S6K1*^*f/f*^ *Ckmm* mice experienced significantly increased latency to fall and maximum speed reached compared to *Lmna*^*−/−*^ *S6K1*^*+/+*^ mice at 5 weeks of age. (**b**) Signaling through the mTORC1 pathway, indicated by p-S6, in muscle (gastrocnemius) tissue of long-lived *Lmna*^*−/−*^ *S6K1*^*f/f*^ *Ckmm* mice. Relative p-S6 levels (normalized to S6) and relative PGC-1α, Cox IV, NRF1 and mtTFA protein levels (normalized to GAPDH) were quantified. Each value is mean±s.e.m. for replicate numbers indicated in parentheses, and statistical significance was determined by unpaired two-tailed Student’s *t-*test.

Consistent with whole-body heterozygosity for *S6K1* in *Lmna*^*−/−*^ mice (*Lmna*^*−/−*^ *S6K1*^*+/−*^) (Figure 2), the long-lived *Lmna*^*−/−*^ *S6K1*^*f/f*^ *Ckmm* mice also have improved muscle function at 5 weeks of age, as evaluated by latency to fall and maximum speed on rotarod (Figure 4a). At the molecular level, PGC-1α and Cox IV were also rescued in muscle tissue (Figure 4b). This improved mitochondrial function in skeletal muscle of long-lived *Lmna*^*−/−*^ *S6K1*^*f/f*^ *Ckmm* mice is also further supported by a significant increased NRF1 and a trend toward increased mtTFA (Figure 4b). Thus, *Lmna*^*−/−*^ *S6K1*^*f/f*^ *Ckmm* mice resemble *Lmna*^*−/−*^ mice bearing the whole-body knockdown *S6K1* (*Lmna*^*−/−*^ *S6K1*^*+/−*^ mice; Figure 2) for lifespan extension and improved muscle function. Consistent with findings in the *Lmna*^*−/−*^ *S6K1*^*+/−*^ mice, we did not observe a significant suppression of p-S6 in muscle of *Lmna*^*−/−*^ *S6K1*^*f/f*^ *Ckmm* mice (Figure 4b) even though S6K1 levels were reduced as expected (Figure 3a).

Of note, the partial knockdown S6K1 is also observed in the heart tissue of long-lived *Lmna*^*−/−*^ *S6K1*^*f/f*^ *Ckmm* mice (Figure 3a), reflecting previously published data showing that Cre driven by the muscle creatine kinase promoter is also partially expressed in cardiac tissue [28]. This raised a caveat that extended survival of *Lmna*^*−/−*^ *S6K1*^*f/f*^ *Ckmm* mice may be attributable to improved cardiac function due to genetic ablation of *S6K1*. However, the rescue of PGC-1α and Cox IV protein levels in muscle tissue (Figure 4b) were not observed in heart tissue of long-lived *Lmna*^*−/−*^ *S6K1*^*f/f*^ *Ckmm* mice (Supplementary Figure S8). In addition, even though long-lived *Lmna*^*−/−*^ *S6K1*^*+/−*^ mice had reduced levels of phospho-rpS6 in cardiac tissue (Supplementary Figure S5B), no improvement of cardiac function was observed (Supplementary Figure S5A) and PGC-1α protein levels were unaffected (Supplementary Figure S5B).

### Whole-body overexpression of 4E-BP1 shortened survival of *Lmna*^*−/−*^ mice

Another well-studied downstream target of mTORC1 is the translational repressor, 4E-BP1 [2, 3], the eIF4E-binding protein. If rapamycin extends lifespan of *Lmna*^*−/−*^ by reducing mTORC1-mediated phosphorylation of 4E-BP1 [15], *Lmna*^*−/−*^ mice bearing whole-body 4E-BP1-overexpression might be expected to live longer than *Lmna*^*−/−*^ mice and have reduced pathology. To test this hypothesis, we crossed *Lmna*^*+/−*^ mice with those expressing whole-body 4E-BP1 [29]. to generate *Lmna*^*−/−*^ mice overexpressing 4E-BP1 (*Lmna*^*−/−*^ *4E-BP1*) (Supplementary Figure S1C).

**Figure 5:**
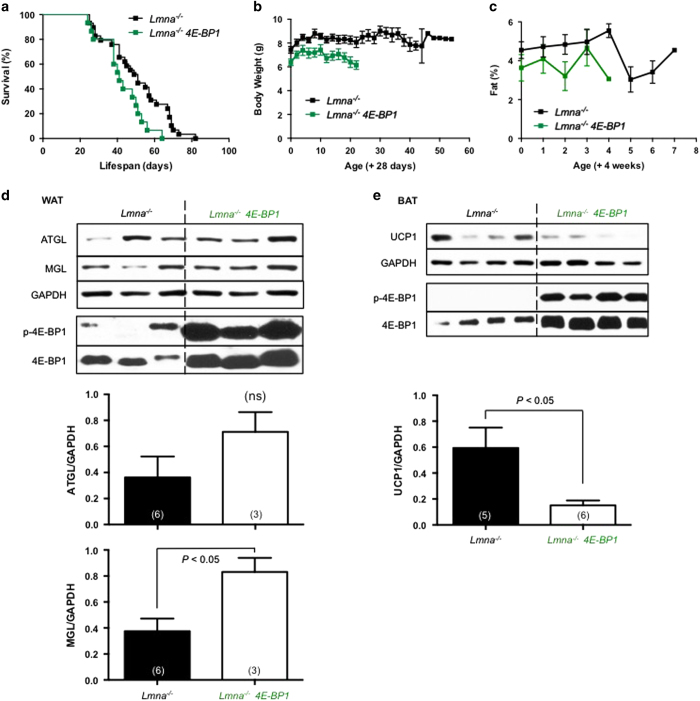
Overexpression of 4E-BP1 shortened survival of *Lmna*^*−/−*^ mice. Genetically overexpressing 4E-BP1 shortens/impairs survival in *Lmna*^*−/−*^ mice. (**a**) Kaplan–Meier survival plot of *Lmna*^*−/−*^ (*n*=29, black) and *Lmna*^*−/−*^ *4E-BP1* (*n*=15, green) mice. Survival is significantly decreased in *Lmna*^*−/−*^ *4E-BP1* mice (*P*=0.0174 by log-rank test), resulting in a 19% decrease in mean lifespan (50.5 vs 42.5 days). Data from males and females are combined. Symbols represent individual mice. (**b**) Body weight (BW) of *Lmna*^*−/−*^ (started with *n*=24, black) and *Lmna*^*−/−*^ *4E-BP1* (started with *n*=13, green) mice were measured every other day started at 4 weeks of age. (**c**) Adiposity (percent body fat) was measured weekly ((fat mass/BW)×100) from *Lmna*^*−/−*^ (started with *n*=17, black) and *Lmna*^*−/−*^ *4E-BP1* (started with *n*=12, green) mice. (**d**) Activity of lipolysis, indicated by ATGL and MGL, in white adipose tissue (WAT) of *Lmna*^*−/−*^ (*n*=6) and *Lmna*^*−/−*^ *4E-BP1* (*n*=3) mice. Relative ATGL and MGL levels (normalized to GAPDH) were quantified. (**e**) Western blots of UCP1 levels in brown adipose tissue (BAT) derived from *Lmna*^*−/−*^ (*n*=5) and *Lmna*^*−/−*^ *4E-BP1* (*n*=6) mice. Relative UCP1 levels (normalized to GAPDH) were quantified. Each value is mean±s.e.m. for replicate numbers indicated in parentheses, and statistical significance was determined by unpaired two-tailed Student’s *t-*test. ns, no significance.

To our surprise, ubiquitous overexpression of 4E-BP1 shortened the mean lifespan of *Lmna*^*−/−*^ mice by 19% (Figure 5a). With respect to the sex of the mice, *Lmna*^*−/−*^ *4E-BP1* female mice are more adversely affected than males (Supplementary Figure S9A, D). This short lifespan may relate to extremely small body size of the *Lmna*^*−/−*^*4E-BP1* mice (Figure 5b and c, Supplementary Figure S9B, C, E, F), a finding consistent with our previous study showing that wild-type mice overexpressing 4E-BP1 have smaller body size and less adiposity [29].

We evaluated lipolysis in WAT of *Lmna*^*−/−*^ *4E-BP1* mice, as measured by levels of ATGL and monoacylglycerol lipase (MGL), as well as thermogenesis, as indicated by levels of UCP1 in BAT. Surprisingly, lipolysis was further elevated in WAT and thermogenesis was further suppressed in BAT of *Lmna*^*−/−*^ *4E-BP1* mice (Figure 5d and e). Increased lipolysis and suppressed thermogenesis suggest that *Lmna*^*−/−*^ *4E-BP1* mice may experience further increased energy expenditure. This *Lmna*^*−/−*^ *4E-BP1* mouse model also further supports our hypothesis that elevated energy expenditure is one factor that shortens the survival of *Lmna*^*−/−*^ mice [16].

In summary, overexpression of 4E-BP1 exaggerates the small phenotype in *Lmna*^*−/−*^ mice and further enhances early mortality. These results also echo our previous study that rapamycin did not affect phosphorylation nor total levels of 4E-BP1 protein in *Lmna*^*−/−*^ mice, especially in heart and muscle tissues [15]. Thus, all these results further suggest that rapamycin extends survival of *Lmna*^*−/−*^ mice at least in part by mediating the mTORC1-S6K1 branch of the pathway, but not the mTORC1-4E-BP1 branch.

## Discussion

Rapamycin-mediated mTORC1 inhibition rescues cardiac, skeletal muscle and adipose function and robustly enhances survival in *Lmna*^*−/−*^ mice [15, 16], a model for the cardiomyopathy and muscular dystrophy associated with human mutations in *LMNA* [8]. Here, we identified a key molecular mechanism underlying lifespan extension by rapamycin in *Lmna*^*−/−*^ mice. Specifically, genetically ablation of *S6K1*, a downstream of mTORC1, in *Lmna*^*−/−*^ mice (*Lmna*^*−/−*^ *S6K1*^*+/−*^ mice) resembles the effect of rapamycin in *Lmna*^*−/−*^ mice. This improved survival in long-lived *Lmna*^*−/−*^ *S6K1*^*+/−*^ mice is not due to improved function in cardiac nor adipose tissues, but likely skeletal muscle given the fact that genetic ablation of *S6K1* specifically in muscle tissues (*Lmna*^*−/−*^ *S6K1*^*f/f*^ *Ckmm* mice) also improved survival of *Lmna*^*−/−*^ mice. Lifespan extension by contrast is not observed in *Lmna*^*−/−*^ mice overexpressing 4E-BP1, the other canonical downstream of mTORC1. Thus, rapamycin extends survival of *Lmna*^*−/−*^ mice at least by suppressing S6K1 pathway in muscle (Supplementary Figure S10).

We found that reducing S6K1 activity improved the survival of *Lmna*^*−/−*^ mice, likely by improving muscle function and possibly by rescuing PGC-1α protein levels in skeletal muscle. Given that PGC-1α is a master co-transcriptional factor regulator of mitochondrial biogenesis and mitochondrial function, [22, 30]. which declines with age [31], this is likely a critical factor underlying muscle-specific defects in *Lmna*^*−/−*^ mice. Increased PGC-1α expression in long-lived *Lmna*^*−/−*^ mice (*Lmna*^*−/−*^ *S6K1*^*+/−*^ and *Lmna*^*−/−*^ *S6K1*^*f/f*^ *Ckmm* mice) presumably induces mitochondrial subunits Cox IV and drives more ATP generation. Consistently, PGC-1α expression and ATP production are reduced in fibroblasts derived from Hutchinson-Gilford progeria syndrome, a lethal genetic disease caused by point mutation in *LMNA*, and both can be rescued by methylene blue, a mitochondrial-targeting antioxidant [32]. Of note, long-lived *S6K1*^*−/−*^ mice display improved rotarod performance [18] as well as elevated PGC-1α expression in both muscle and adipose tissues [33]. Life extension by dietary restriction, which also suppresses S6K1 signaling, improves rotarod performance in mice [34] and increases both PGC-1α and Cox IV expression in skeletal muscle [35]. Furthermore, PGC-1α is also required for the dietary restriction-induced increases in mitochondrial gene expression and mitochondrial density in skeletal muscle [36]. These independent studies, coupled with our findings, further demonstrate the conserved roles of mTORC1-S6K1 signaling in normal aging and *Lmna* gene-derived laminopathies, particularly with respect to muscle function.

Although reducing mTORC1-S6K1 activity improves muscle function and extends lifespan in both normal and *Lmna*^*−/−*^ mice, many studies show that intact mTORC1-S6K1 signaling is required for muscle function. For instance, muscle-specific inactivation of mTOR leads to severe myopathy, resulting in premature death [37]. Skeletal muscle-specific ablation of raptor (mTORC1) can cause metabolic changes, reduces mitochondrial biogenesis and results in muscle dystrophy [38]. During muscle hypertrophy, S6K1 is required for skeletal muscle force production [39]. Furthermore, mice deficient in ribosomal protein S6 phosphorylation (rpS6^P*−/−*^ mice), a downstream target of S6K1, suffer from muscle weakness that reflects a growth defect and energy deficit [40]. These studies based on genetic mouse models indicate that mTORC1 pathway is a crucial regulator of skeletal muscle growth and function. Notably, mTORC1 signaling is aberrantly elevated in skeletal muscle in *Lmna*^*−/−*^ mice [15], and the reduced mTORC1 activity in *Lmna*^*−/−*^ *S6K1*^*+/−*^ mice may compensate for this aberrant elevation. Double knockout (*Lmna*^*−/−*^ *S6K1*^*−/−*^) mice may not be long-lived because of effects in other tissues. For instance, these mice exhibit an even greater reduction in BW (Supplementary Figure S2H, I), which is likely deleterious to survival in an already cachexic background (Supplementary Figure S2G). Together, these findings suggest that the levels of mTORC1 activity must be well-balanced—too much contributes to pathology in disease states (and possibly normal aging), whereas too little impairs the ability of tissues to respond to stress and/or regenerate.

The role of mTORC1-S6K1 in cardiovascular aging is well documented [41, 42]. Specifically, suppression of mTORC1-S6K1 signaling, either by rapamycin or dietary restriction, improves cardiac functions in normal mice [43–45]. Previously, we also found that suppression of S6K1 signaling by rapamycin improves cardiac function in *Lmna*^*−/−*^ mice [15]. Intriguingly, improved cardiac function was not observed in long-lived *Lmna*^*−/−*^ *S6K1*^*+/−*^ mice (Supplementary Figure S5). Although it may be possible that complete inhibition of S6 kinase activity is required for restoration of cardiac function, it is also possible that the pathology evoked by aberrant mTORC1 signaling in this tissue occurs through phosphorylation of other mTORC1 substrates. We have previously shown that autophagy is impaired in heart tissue of *Lmna*^*−/−*^ mice and speculate that this may be mediated through phosphorylation of ULK1, or other autophagy-related substrates [15].

It has been suggested that the regulation of mTORC1-mediated fat metabolism involves signaling through S6K1 and 4E-BP1 [46–48]. More direct evidence of mTORC1-S6K1 pathway’s role in fat metabolism has been obtained from *S6K1*^*−/−*^ mice [18, 33], where a lean phenotype and resistance to obesity in part owing to increased lipolysis [33]. Furthermore, mice with adipose-specific deficiency of raptor, an mTORC1 component, show a lean phenotype, which is related to increased energy expenditure and increased mitochondrial uncoupling [49]. However, we did not observe a significant difference in the adiposity of long-lived *Lmna*^*−/−*^ *S6K1*^*+/−*^ and *Lmna*^*−/−*^ *S6K1*^*f/f*^ *Ckmm* mice compared with controls. Thus, altered adiposity may not be linked to enhanced survival in *Lmna*^*−/−*^ *S6K1*^*+/−*^ and *Lmna*^*−/−*^ *S6K1*^*f/f*^ *Ckmm* mice [16].

To our surprise, overexpression of 4E-BP1 shortened the survival of *Lmna*^*−/−*^ mice, especially in females (Figure 5, Supplementary Figure S9). The results are unexpected, considering the beneficial effects of 4E-BP1 in metabolism of wild-type mice. For instance, wild-type mice with increased 4E-BP1 expression are resistant to high-fat diet-induced obesity [29]. Conversely, the amount of WAT is significantly decreased in male *4E-BP1*^*−/−*^ mice [50]. Combined genetic ablation of *4E-BP1* and *4E-BP2*, that is, hyperactivation of mTORC1 signaling throughout body (*4E-BP1 and 2*^*−/−*^ mice), also led to decreased lipolysis, increased TGA accumulation, and increased insulin resistance [51]. A follow-up study showed that mouse embryonic fibroblasts derived from mice lacking 4E-BPs accumulate more fat by suppressing ATGL, the enzyme involved in the first step of triglyceride hydrolase activity in lipolysis [52]. In line with this regulatory mechanism, short-lived *Lmna*^*−/−*^ *4E-BP1* mice also experienced elevated ATGL in WAT (although only a trend) as well as decreased UCP1 in BAT (Figure 5). Thus, we speculate that the deleterious effect of 4E-BP1 overexpression in *Lmna*^*−/−*^ mice might be a result of further increased energy expenditure, causing a reduction in adiposity and a shorter lifespan [16]. In sum, these findings indicate that altered mTORC1-4E-BP1 signaling cannot explain rapamycin-mediated life extension in *Lmna*^*−/−*^ mice.

Based on results from our genetic interventions, the life extension by rapamycin in *Lmna*^*−/−*^ mice is likely mediated by suppressing mTORC1-S6K1 signaling, which improves mitochondrial activity by rescuing the suppressed PGC-1α expression in skeletal muscle. However, mTORC1-4E-BP1 signaling may not be involved in lifespan extension. It appears that 4E-BP1 overexpression may reduce the adiposity in already lipoatrophic *Lmna*^*−/−*^ mice. Although a reduction in S6K1 activity leads to a 20~30% lifespan extension in *Lmna*^*−/−*^ mice, modulation of S6K1 cannot recapitulate all the beneficial effects by rapamycin, which doubles their survival [15, 16]. This suggests that mTORC1 phosphorylation of multiple substrates may account for different aspects of toxicity. As stated the cardiac toxicity may reflect mTORC1-mediated control of autophagy [15] and the adipose phenotypes may be mediated through inhibition of UCPs in BAT [46]. Therefore, to recapitulate the effects of rapamycin, it may be necessary to target multiple downstream substrates. We posit that from a therapeutic perspective, with respect to laminopathies, other disease and possibly aging itself, it may be necessary for full efficacy to target mTORC1 directly, or perhaps upstream components of the signaling pathway. Furthermore, human diseases mimicking *Lmna*^*−/−*^ mice—muscular dystrophy and progeroid syndromes—targeted inhibition of S6K1 might be an effective therapeutic approach, whereas not as powerful as rapamycin but might limit associated immunosuppressive side effects.

## Materials and methods

### Mice husbandry

Mice were bred and maintained under specific pathogen-free conditions. A tail biopsy was performed in weaning mice at 3 weeks of age for genotyping by polymerase chain reaction with specific primers. All the animals had food and water *ad libitum* and were kept in standard temperature conditions (22 °C) and 12:12-h light–dark cycles. All animal care and experimental procedures were approved by the Institutional Animal Care and Use Committee at the Buck Institute for Research on Aging.

### Generation of whole-body S6K1 knockdown mice in *Lmna*^*−/−*^ context

*Lmna*^*+/−*^ mice (C57BL/6J genetic background) [15] were crossed with *S6K1*^*+/−*^mice (C57BL/6J genetic background) [17] to generate heterozygotic (*Lmna*^*+/−*^ *S6K1*^*+/−*^) mice (Supplementary Figure 1A). Male double heterozygotic (*Lmna*^*+/−*^ *S6K1*^*+/−*^) mice and female double heterozygotic (*Lmna*^*+/−*^ *S6K1*^*+/−*^) mice were mated to produce *Lmna*^*−/−*^ *S6K1*^*+/−*^, *Lmna*^*−/−*^ *S6K1*^*−/−*^ and wild-type mice (*Lmna*^*+/+*^ *S6K1*^*+/+*^) for the present experiment.

### Generation of muscle-specific *S6K1* knockdown mice in *Lmna*^*−/−*^ context

*Lmna*^*+/−*^ mice (C57BL/6J genetic background) [15] were crossed with mice bearing one floxed *S6K1* allele mice (*S6K1*^*flox/+*^ or *S6K1*^*f/+*^) (C57BL/6J genetic background) [27] to generate *Lmna*^*+/−*^ *S6K1*^*f/+*^ mice (Supplementary Figure 1B). Meanwhile, *S6K1*^*f/+*^ mice were crossed with mice expressing Cre recombinase under the control of the muscle creatine kinase promoter (*Ckmm-Cre*) (C57BL/6J genetic background) [28], yielding *S6K1*^*f/+*^ *Ckmm* mice. *Lmna*^*+/−*^ *S6K1*^*f/+*^ *Ckmm* mice were then generated by crossing *Lmna*^*+/−*^ mice with *S6K1*^*f/+*^ *Ckmm* mice. *Lmna*^*+/−*^ *S6K1*^*f/+*^ mice and *Lmna*^*+/−*^ *S6K1*^*f/+*^ *Ckmm* mice were subsequently crossed to generate the following genotypes: *Lmna*^*−/−*^ *S6K1*^*+/+*^, *Lmna*^*−/−*^ *S6K1*^*f/+*^ *Ckmm* and *Lmna*^*−/−*^ *S6K1*^*f/f*^ *Ckmm* mice.

### Generation of *Lmna*^*−/−*^ mice overexpressing 4E-BP1

*Lmna*^*+/−*^ mice (C57BL/6J genetic background) [15] were crossed with mice overexpressing one copy of *4E-BP1* (C57BL/6J genetic background), which is described in our previous study[29]. (Supplementary Figure 1C). Then, *Lmna*^*+/−*^ mice were crossed with *Lmna*^*+/−*^ *4E-BP1* mice to generate *Lmna*^*−/−*^ mice as well as *Lmna*^*−/−*^ *4E-BP1* mice for the present experiment.

### Lifespan study

All mice on the lifespan studies were monitored everyday from 4-week of age until the end of life. BW was measured every other day in all mice. No mice used for the lifespan study were used for any other biochemical or metabolic tests.

### Body composition

Whole-body composition (fat mass, lean mass and free water) analysis was conducted weekly using quantitative nuclear magnetic resonance machine (EchoMRI-2012; Echo Medical Systems, Houston, TX, USA) starting at 4 weeks of age.

### Rapamycin injection

Mice were injected intraperitoneally with 8 mg kg^−1 ^ BW rapamycin (LC Laboratories, Woburn, MA, USA) or vehicle every other day according to our previous study [16]. A stock solution of 50 mg ml^−1^ rapamycin was prepared in 100% ethanol and stored at −20 °C. Rapamycin was then diluted in vehicle (5% polyethylene glycol and 5% Tween 80) before injection. The vehicle control consisted of the same volume ethanol.

### GTT

GTT performed on non-anesthetized animals. Mice were fasted with access to water for 16 h (overnight) before being given a single injection intraperitoneally with 20% glucose at a dose of 2 g kg^−1 ^of BW. The tail prick was used for blood glucose measurement at time points 0, 30, 60, 90, 120 and 180 min with an ACCU-CHEK Aviva glucometer (Roche Diagnostics, Dallas, TX, USA) and the test strips. The mice for rapamycin study were on 129Sv-C57BL/6J genetic background [16].

### Transthoracic echocardiography

Mouse cardiac structure and contractility were imaged using VisualSonics Vevo2100 at 5–6 weeks of age. These experimental approaches were adapted from our previous study [15, 43, 53]. In brief, mice were initially anesthetized with ~2.25% isofluorane, placed on a heating pad (37 °C) and maintained under light anesthesia so as to maintain the highest possible heart rate during data collection. LV parameters were obtained from M-mode recordings. LV end diastolic diameter and systolic diameter were calculated from the mean of at least three separate cardiac cycles. To calculate myocardial performance index and the E/A ratio, pulsed wave Doppler measurements were taken in the four-chamber view of the heart. All standard cardiac parameters were calculated off-line using VisualSonics software (v1.3.0).

### Rotarod

In forced motor activity on a rotating rod (rotarod) assays to assess motor and neurological function was adapted from our previous study [15]. In brief, the day before the actual rotarod testing, the mice were placed on the rotarod set to a beginning speed of 5 r.p.m., with an acceleration rate of 0.1 r.p.m. s^−1^. The max speed was set at 80 r.p.m.. They were allowed to practice the rotarod five times, one repetition every 5 min. If the animal did not fall off, each repetition would end at 5 min. The animals were tested 24 h after the practice day, and the procedure was the same except scores were recorded. The score for each repetition was the time in second and the speed in r.p.m. until the animal fell off the rotarod. The average of five repetitions was used to score the sessions.

### Tissue harvesting and immunoblotting

Tissues were dissected from the mice and immediately frozen in liquid nitrogen for western blotting analysis. Muscle (gastrocnemius), heart, liver, WAT and BAT were harvested and immediately frozen in liquid nitrogen. Tissue samples were lysed in cold RIPA buffer supplemented with phosphatase inhibitor and protease inhibitor cocktail tablets. Tissue sections were homogenized using the Omni TH homogenizer (Omni International, Kennesaw, GA, USA) on ice in RIPA buffer (300 mM NaCl, 1.0% NP-40, 0.5% sodium deoxycholate, 0.1% SDS, 50 mM Tris (pH 8.0), protease inhibitor cocktail (Roche) and phosphatase inhibitor 2, 3 (Sigma, Atlanta, GA, USA)) and then centrifuged at 13 200 r.p.m. for 10 min at 4 °C. The supernatants were collected and protein concentrations were determined using the DC protein assay (Bio-Rad, Los Angeles, CA, USA). Equal amounts of protein were resolved by SDS-PAGE (4–12% Bis-Tris gradient gel, Invitrogen, Swedesboro, NJ, USA), transferred to membranes and analyzed by western blotting with protein-specific antibodies. The antibodies against the phosphorylated rsS6^S240/244^ (5364), S6 (2217), ATGL (2439), the phosphorylated 4E-BP1^S65^ (9451), 4E-BP1 (9452), the phosphorylated HSL^S563^ (4139), S6K1 (2708), FAS (3180), Cox IV (4850), NRF1 (46743) and glyceraldehyde 3-phosphate dehydrogenase (2118) were purchased from Cell Signaling Technology (Danvers, MA, USA). PGC-1α (ab54481), monoacylglycerol lipase (ab24701) and UCP1 (ab23841) were purchased from Abcam (Boston, MA, USA). Desmin (sc23879) and mtTFA (sc166965) were purchased from Santa Cruz Biotechnology (Santa Cruz, CA, USA). Protein bands were revealed using the Amersham ECL detection system (GE Healthcare, Marlborough, MA, USA) and quantified by densitometry using ImageJ software (http://rsb.info.nih.gov/ij/).

### Statistical analysis

All statistical analyses were conducted using GraphPad Prism 6 (GraphPad, La Jolla, CA, USA). The survival curves were completed using a Kaplan–Meier curve. We used a log-rank (Mantel–Cox) test to perform the statistical analyses of the survival curves. All the other data are shown as mean±s.e.m. The statistical significance of differences between two groups was determined using unpaired, two-tailed Student’s *t*-test.
