# Supplementary material for: Evidence that S6K1, but not 4E-BP1, mediates skeletal muscle pathology associated with loss of A-type lamins
Source: Cell Discov. 2017 Oct 31;3:17039–. doi: 10.1038/celldisc.2017.39 (PMC5931234; doi:10.1038/celldisc.2017.39)
Supplement: Supplementary Information [file celldisc201739-s1.pdf]

**SUPPLEMENTARY INFORMATION**

**FIGURE S1**

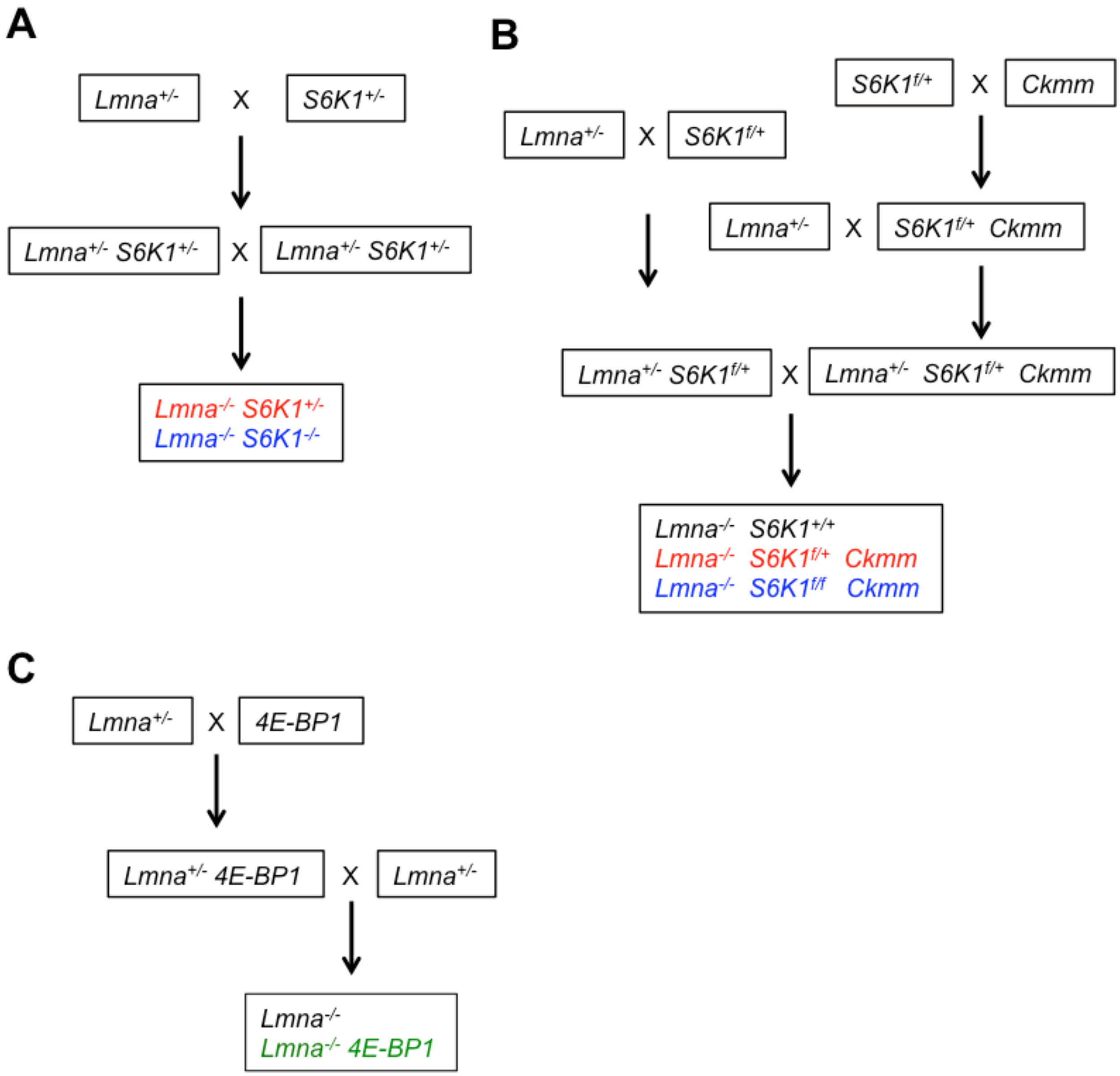


**Supplementary Figure S1. Breeding strategies for generating genetic ablation of *S6K1* or overexpression of *4E-BP1* in *Lmna*$^{-/-}$ mice, related to Figure 1, 3 and 5.** (**A**) Schematic crossing strategies for breeding *S6K1* knockdown/knockout in the context of *Lmna*$^{-/-}$ mice (*Lmna*$^{-/-}$ *S6K1*$^{+/-}$ and *Lmna*$^{-/-}$ *S6K1*$^{-/-}$). (**B**) Schematic crossing strategies for breeding muscle-specific *S6K1* knockout in the context of *Lmna*$^{-/-}$ mice from *Lmna*$^{+/-}$ mice, mice bearing one allele floxed *S6K1* gene (*S6K1*$^{flox/+}$ or *S6K1*$^{f/+}$ mice), and mice expressing Cre recombinase under the control of the muscle creatine kinase promoter (Ckmm mice). (**C**) Schematic crossing strategies for breeding *4E-BP1*-overexpression in the context of *Lmna*$^{-/-}$ mice (*Lmna*$^{-/-}$ *4E-BP1*).

**FIGURE S2**

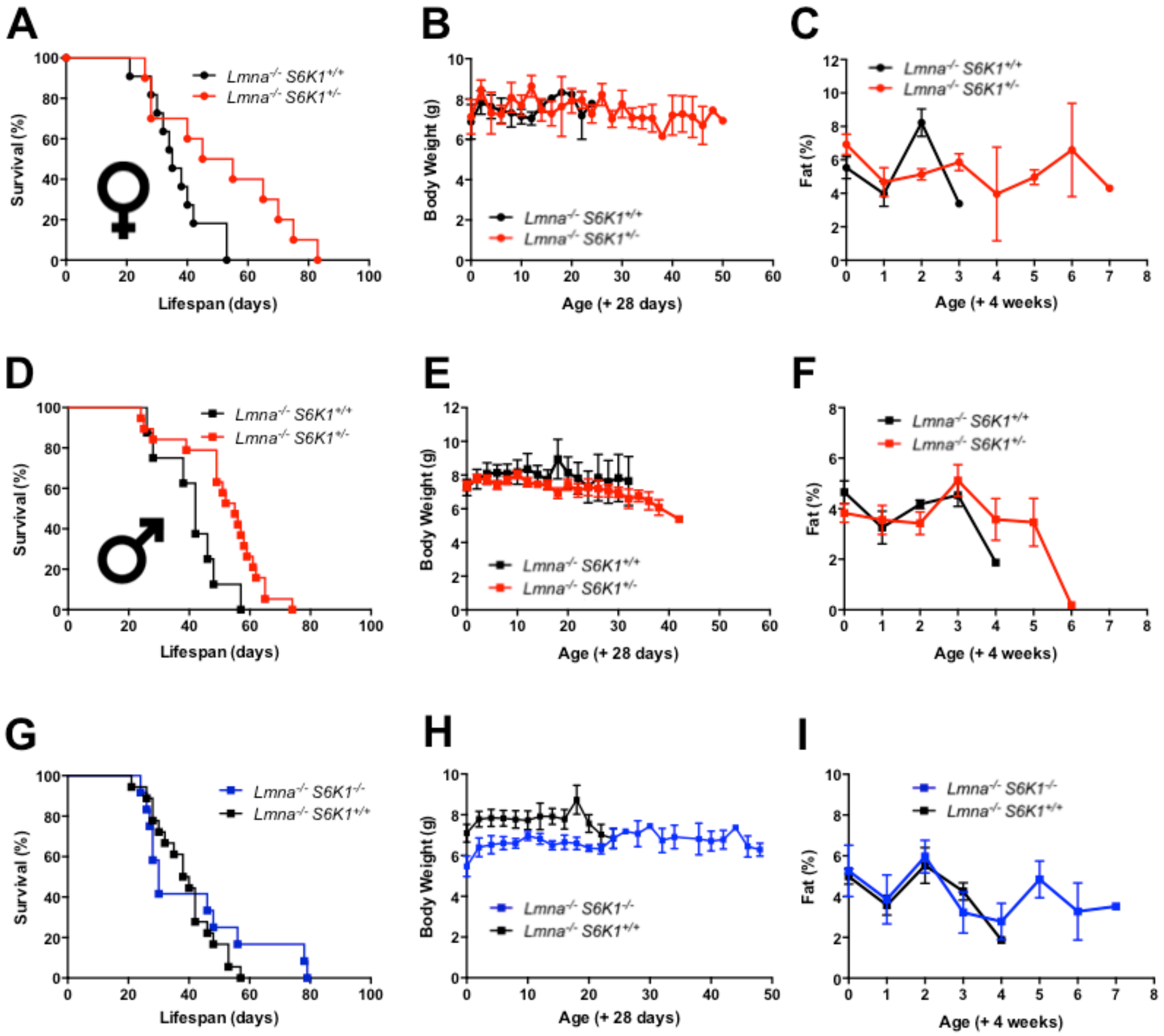


**Supplementary Figure S2. Survivorship of genetic ablation of S6K1 in *Lmna*$^{-/-}$ mice, related to Figure 1.** (**A**) Kaplan-Meier survival plot of female *Lmna*$^{-/-}$ *S6K1*$^{+/+}$ (n = 11, black) and *Lmna*$^{-/-}$ *S6K1*$^{+/-}$ (n = 10, red) mice. Symbols represent individual mice. (**B**) Body weight (BW) of female *Lmna*$^{-/-}$ *S6K1*$^{+/+}$ (started with n = 5, black) and *Lmna*$^{-/-}$ *S6K1*$^{+/-}$ (started with n = 5, red) mice. (**C**) Adiposity (percent body fat) was measured weekly ([fat mass/BW] x 100) from female *Lmna*$^{-/-}$ *S6K1*$^{+/+}$ (started with n = 5, black) and *Lmna*$^{-/-}$ *S6K1*$^{+/-}$ (started with n = 8, red) mice. (**D**) Kaplan-Meier survival plot of male *Lmna*$^{-/-}$ *S6K1*$^{+/+}$ (n = 8, black) and *Lmna*$^{-/-}$ *S6K1*$^{+/-}$ (n = 19, red) mice. Symbols represent individual mice. (**E**) BW of male *Lmna*$^{-/-}$ *S6K1*$^{+/+}$ (started with n = 7, black) and *Lmna*$^{-/-}$ *S6K1*$^{+/-}$ (started with n = 13, red) mice. (**F**) Adiposity (percent body fat) was measured weekly ([fat mass/BW] x 100) from male *Lmna*$^{-/-}$ *S6K1*$^{+/+}$ (started with n = 6, black) and *Lmna*$^{-/-}$ *S6K1*$^{+/-}$ (started with n = 14, red) mice. (**G**) Kaplan-Meier survival plot of *Lmna*$^{-/-}$ *S6K1*$^{+/+}$ (n = 19, black) and double-knockout *Lmna*$^{-/-}$ *S6K1*$^{-/-}$ (n = 12, blue) mice. Data from males and females are combined. Symbols represent individual mice. (**H**) BW of *Lmna*$^{-/-}$ *S6K1*$^{+/+}$ (started with n = 12, black) and *Lmna*$^{-/-}$ *S6K1*$^{-/-}$ (started with n = 8, blue) mice. (**I**) Adiposity (percent body fat) was measured weekly ([fat mass/BW] x 100) from *Lmna*$^{-/-}$ *S6K1*$^{+/+}$ (started with n = 8, black) and *Lmna*$^{-/-}$ *S6K1*$^{-/-}$ (started with n = 6, blue) mice. Data from males and females are combined.

**FIGURE S3**

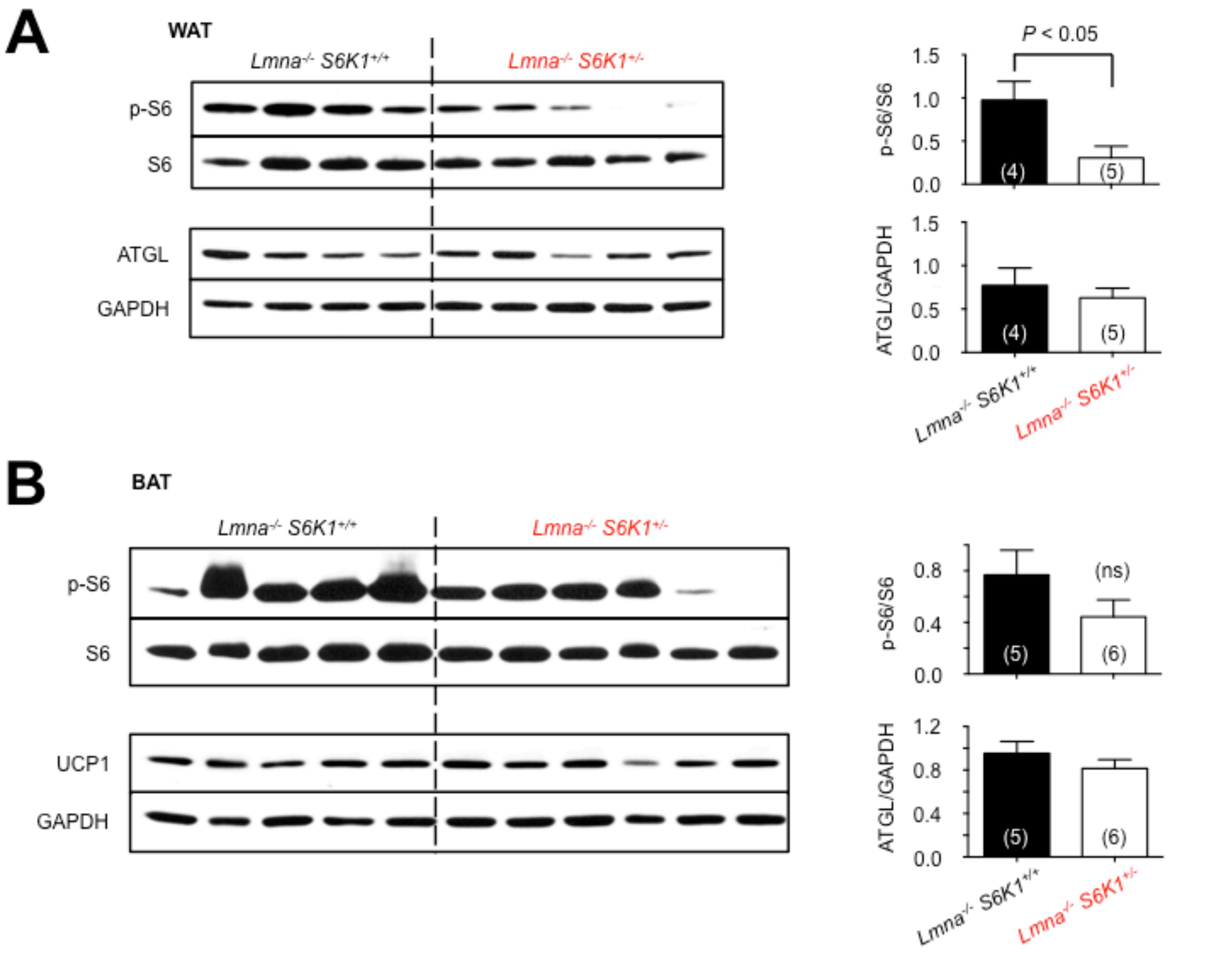


**Supplementary Figure S3. No difference of lipolysis and thermogenesis in long-lived *Lmna*$^{-/-}$ *S6K1*$^{+/-}$ mice.** (**A**) Western blots of mTORC1 activity, indicated by p-S6, in white adipose tissue (WAT) of *Lmna*$^{-/-}$ *S6K1*$^{+/+}$ and *Lmna*$^{-/-}$ *S6K1*$^{+/-}$ mice. Relative p-S6 levels (normalized to S6) were quantified. Relative ATGL levels (normalized to GAPDH) were quantified. (**B**) Western blots of mTORC1 activity, indicated by p-S6, in brown adipose tissue (WAT) of *Lmna*$^{-/-}$ *S6K1*$^{+/+}$ and *Lmna*$^{-/-}$ *S6K1*$^{+/-}$ mice. Relative p-S6 levels (normalized to S6) were quantified. Relative UCP1 levels (normalized to GAPDH) were quantified. Each value is mean ± SEM for number of mice indicated in parentheses, and statistical significance was determined by unpaired two-tailed Student's *t* test. ns, no significance.

**FIGURE S4**

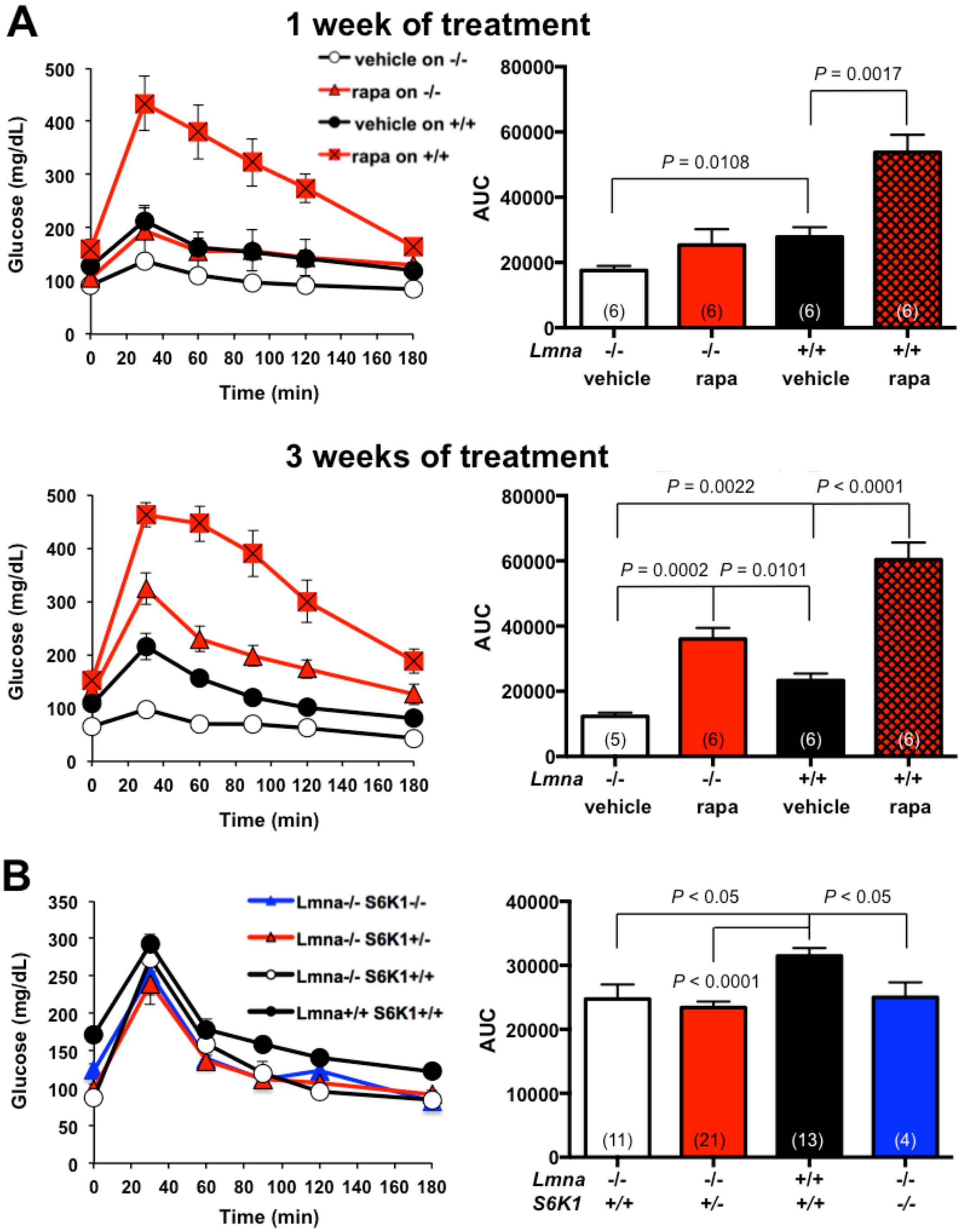


**Supplementary Figure S4. *Lmna* x *S6K1* mice in response to glucose tolerance test.** (**A**) *Lmna*$^{+/+}$ and *Lmna*$^{-/-}$ mice in response to glucose tolerance test (GTT) after 1 or 3 weeks of rapamycin (rapa) treatment. (**B**) *Lmna* x *S6K1* mice in response to GTT at ~5 weeks of age. The area under the curve (AUC) for each GTT is shown on the right. Data from males and females are combined. Each value is mean ± SEM for number of mice indicated in parentheses, and statistical significance was determined by unpaired two-tailed Student's *t* test.

**FIGURE S5**

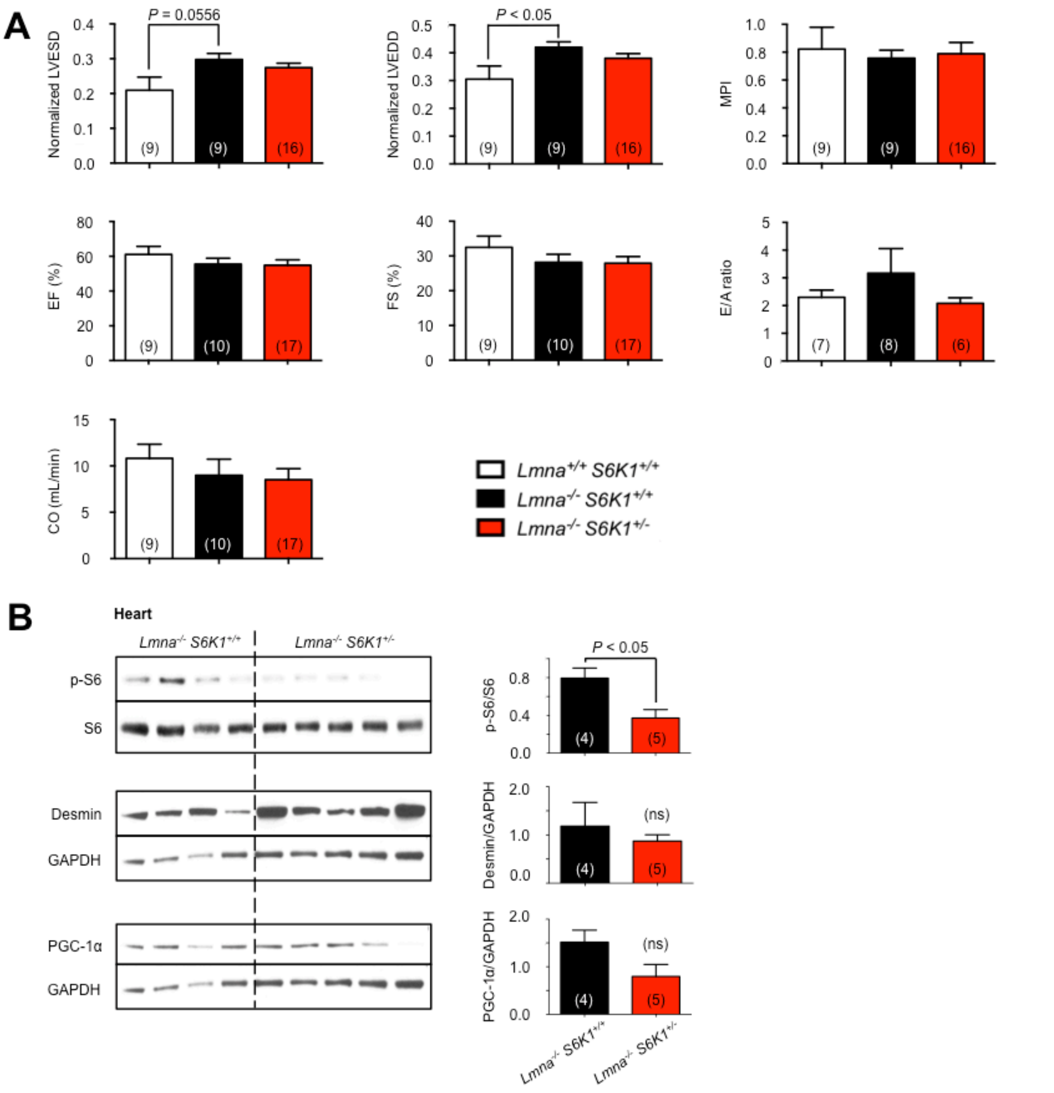


**Supplementary Figure S5. Cardiac function is not improved in long-lived *Lmna*$^{-/-}$ *S6K1*$^{+/-}$ mice.** (**A**) Parameters for cardiac functions of 5~6 weeks old wild type (*Lmna*$^{+/+}$ *S6K1*$^{+/+}$), *Lmna*$^{-/-}$ *S6K1*$^{+/+}$ and *Lmna*$^{-/-}$ *S6K1*$^{+/-}$ mice. **Normalized LVESD** = [LV diameter (mm) / body weight (g)]. **Normal LVEDD** = [LV diameter (mm) / body weight (g)]. LVESD: left ventricular end-systolic diameter. LVEDD: left ventricular end-diastolic diameter. **MPI** = [(IVRT + IVCT) / LVET]. MPI: myocardial performance index. IVRT: isovolumic relaxation time. IVCT: isovolumic contraction time. LVET: left ventricular ejection time. **EF (%)** = [(EDV-ESV)/EDV] x 100. EF: ejection fraction. EDV: end-diastolic volume. ESV: end-systolic volume. **FS (%)** = [(EDD-ESD)/ESD] x 100. FS: fractional shortening. EDD: end-diastolic dimension. ESD: end-systolic dimension. **E/A ratio**. E: mitrial valve E (early) ventricular filling velocity. A: mitrial valve A (late) ventricular filling velocity. **CO**: cardiac output (mL/min). (**B**) Western blots of mTORC1 activity, indicated by p-S6, in heart tissue of *Lmna*$^{-/-}$ *S6K1*$^{+/+}$ and *Lmna*$^{-/-}$ *S6K1*$^{+/-}$ mice. Relative p-S6 levels (normalized to S6) were quantified. Relative desmin and PGC-1α protein level (normalized to GAPDH) was quantified. Each value is mean ± SEM for number of mice indicated in parentheses, and statistical significance was determined by unpaired two-tailed Student's *t* test. ns, no significance.

**FIGURE S6**

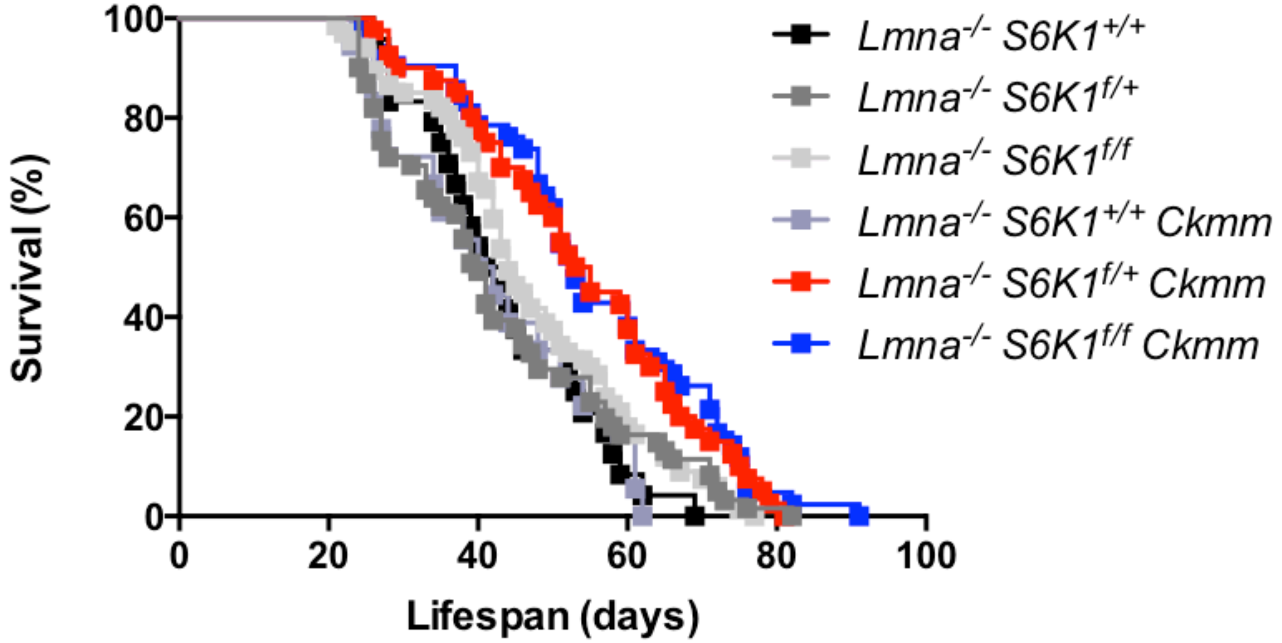


| | *$Lmna^{-/-}$ $S6K1^{f/+}$* (43.11 days, n = 61) | *$Lmna^{-/-}$ $S6K1^{f/f}$* (47.15 days, n = 67) | ***$Lmna^{-/-}$ $S6K1^{+/+}$ Ckmm*** (42.17 days, n = 18) | ***$Lmna^{-/-}$ $S6K1^{f/+}$ Ckmm*** (54.13 days, n = 40) | ***$Lmna^{-/-}$ $S6K1^{f/f}$ Ckmm*** (55.33 days, n = 42) |
|---|---|---|---|---|---|
| ***$Lmna^{-/-}$ $S6K1^{+/+}$*** (43.83 days, n = 24) | *P* = 0.6607 | *P* = 0.1580 | *P* = 0.8564 | ***P* = 0.0016** | ***P* = 0.0006** |
| *$Lmna^{-/-}$ $S6K1^{f/+}$* (43.11 days, n = 61) | | *P* = 0.3396 | *P* = 0.5613 | ***P* = 0.0101** | ***P* = 0.0016** |
| *$Lmna^{-/-}$ $S6K1^{f/f}$* (47.15 days, n = 67) | | | *P* = 0.1694 | ***P* = 0.0121** | ***P* = 0.0047** |
| ***$Lmna^{-/-}$ $S6K1^{+/+}$ Ckmm*** (42.17 days, n = 18) | | | | ***P* = 0.0023** | ***P* = 0.0023** |
| ***$Lmna^{-/-}$ $S6K1^{f/+}$ Ckmm*** (54.13 days, n = 40) | | | | | *P* = 0.5927 |
| ***$Lmna^{-/-}$ $S6K1^{f/f}$ Ckmm*** (55.33 days, n = 42) | | | | | |

**Supplementary Figure S6. Survivorship of *Lmna* x *S6K1* x *Ckmm* mice, related to Figure 3.** Kaplan-Meier survival plot of *$Lmna^{-/-}$ $S6K1^{+/+}$* (black), *$Lmna^{-/-}$ $S6K1^{f/+}$* (steel), *$Lmna^{-/-}$ $S6K1^{f/f}$* (silver), *$Lmna^{-/-}$ $S6K1^{+/+}$ Ckmm* (nickel), *$Lmna^{-/-}$ $S6K1^{f/+}$ Ckmm* (red), and *$Lmna^{-/-}$ $S6K1^{f/f}$ Ckmm* (blue) mice. Data from males and females are combined. Symbols represent individual mice. Mean lifespan in days and number of mice for each genotype are indicated in parentheses. Significant lifespans are indicated in **Bold** by log-rank test.

FIGURE S7

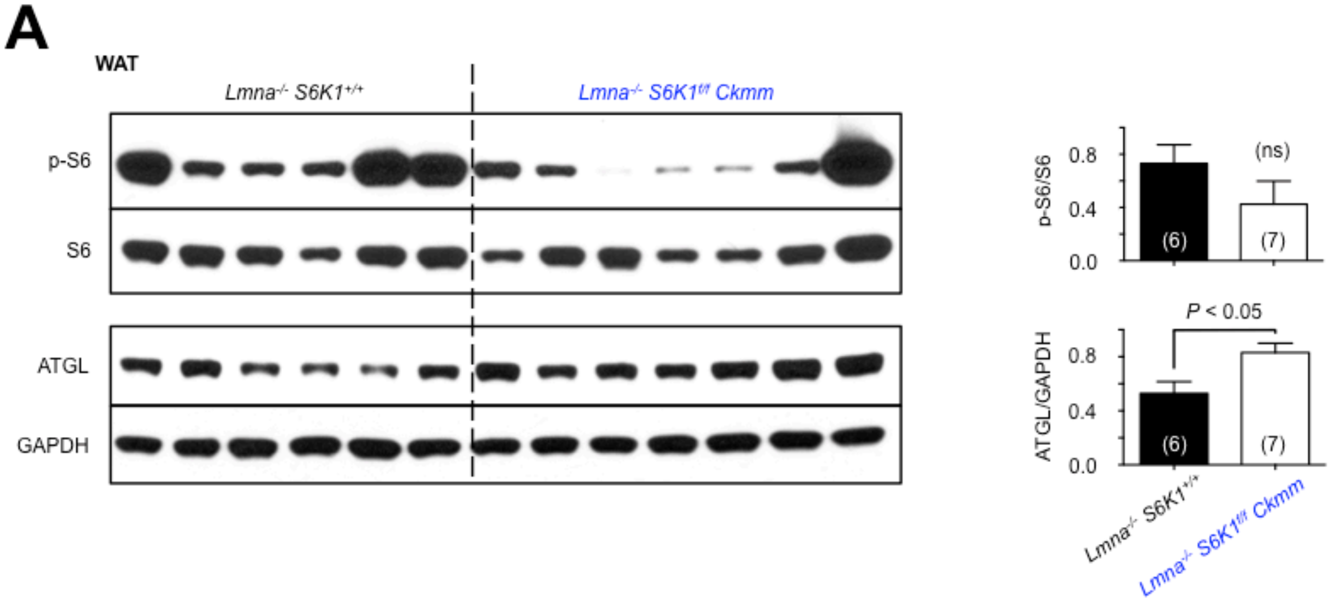


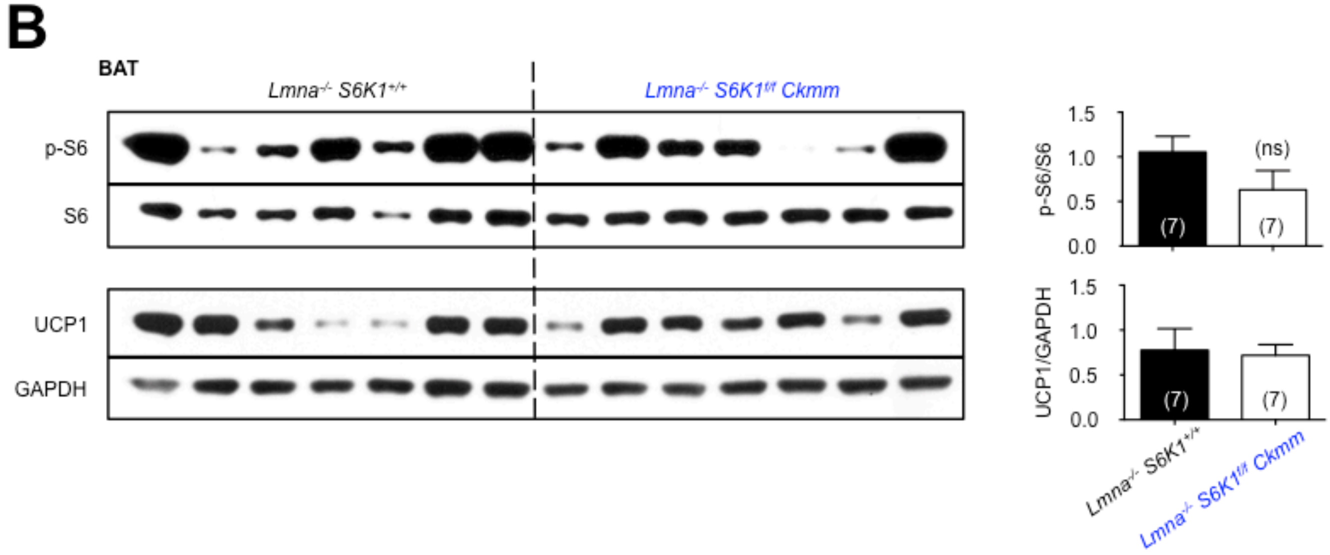


**Supplementary Figure S7. Lipolysis and thermogenesis in long-lived *Lmna*$^{-/-}$ *S6K1*$^{f/f}$ *Ckmm* mice.** (**A**) Western blots of mTORC1 activity, indicated by p-S6, in white adipose tissue (WAT) of *Lmna*$^{-/-}$ *S6K1*$^{+/+}$ and *Lmna*$^{-/-}$ *S6K1*$^{f/f}$ *Ckmm* mice. Relative p-S6 levels (normalized to S6) were quantified. Relative ATGL levels (normalized to GAPDH) were quantified. (**B**) Western blots of mTORC1 activity, indicated by p-S6, in brown adipose tissue (WAT) of *Lmna*$^{-/-}$ *S6K1*$^{+/+}$ and *Lmna*$^{-/-}$ *S6K1*$^{f/f}$ *Ckmm* mice. Relative p-S6 levels (normalized to S6) and UCP1 levels (normalized to GAPDH) were quantified. Each value is mean ± SEM for number of mice indicated in parentheses, and statistical significance was determined by unpaired two-tailed Student's *t* test. ns, no significance.

**FIGUTR S8**

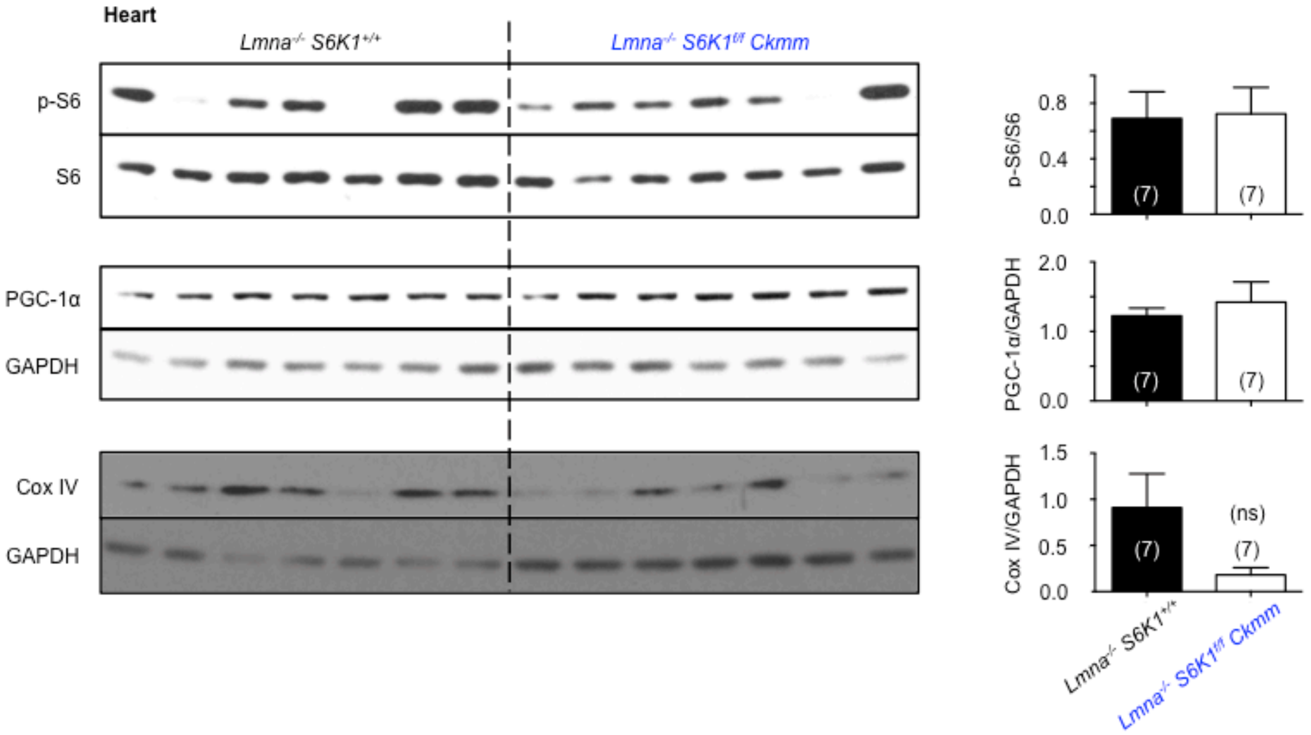


**Supplementary Figure S8. Molecular signaling in heart tissue of long-lived *Lmna$^{-/-}$ S6K1$^{f/f}$ Ckmm* mice.** Western blots of mTORC1 activity, indicated by p-S6, in heart tissue of *Lmna$^{-/-}$ S6K1$^{+/+}$* and *Lmna$^{-/-}$ S6K1$^{f/f}$ Ckmm* mice. Relative p-S6 levels (normalized to S6) and PGC-1α and Cox IV protein levels (normalized to GAPDH) were quantified. Each value is mean ± SEM for number of mice indicated in parentheses, and statistical significance was determined by unpaired two-tailed Student's *t* test. ns, no significance.

**FIGURE S9**

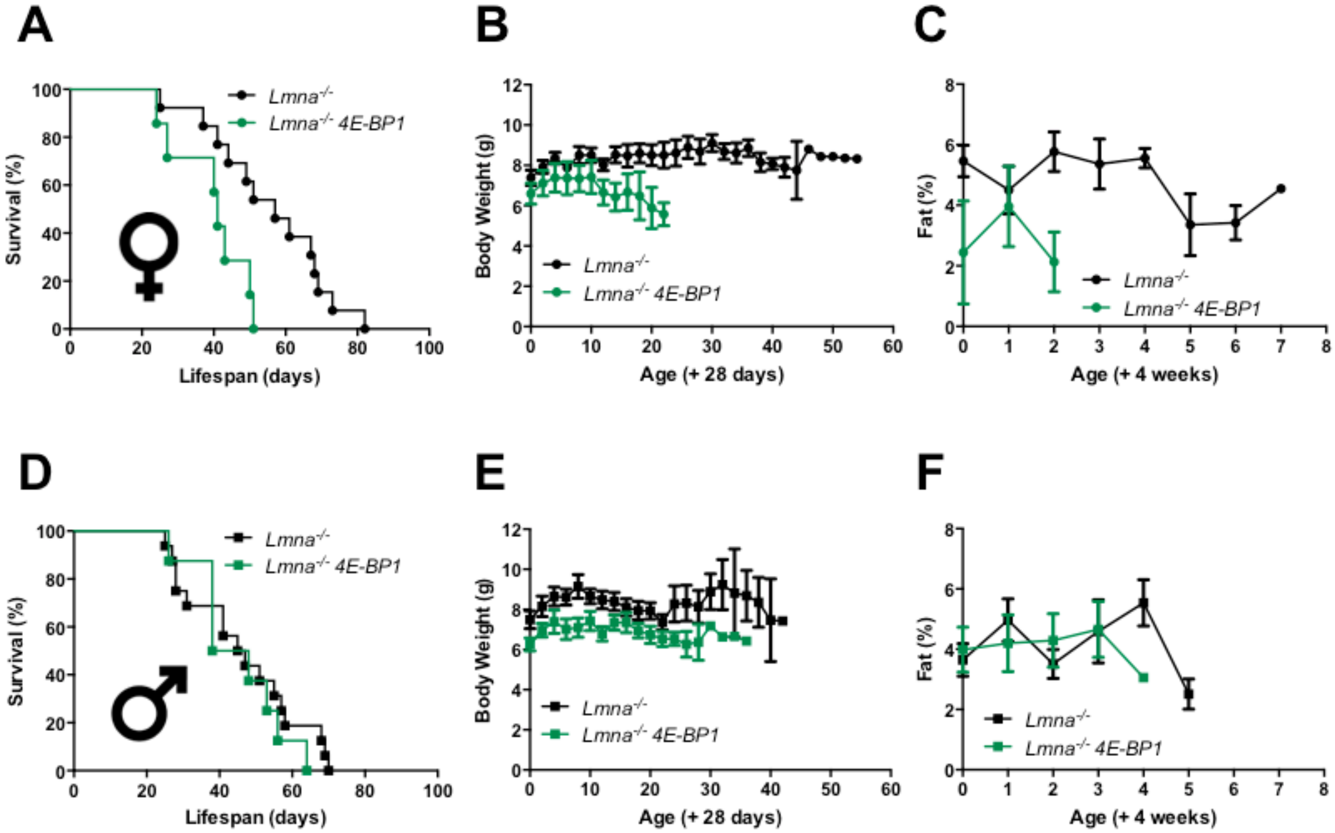


**Supplementary Figure S9. Survivorship of *Lmna*$^{-/-}$ mice overexpressing *4E-BP1*, related to Figure 5.** (**A**) Kaplan-Meier survival plot of female *Lmna*$^{-/-}$ (n = 13, black) and *Lmna*$^{-/-}$ *4E-BP1* (green, n = 7) mice. Symbols represent individual mice. Survival is significantly decreased in female *Lmna*$^{-/-}$ *4E-BP1* mice ($P$ = 0.0101 by log-rank test), resulting in a 29% decrease in mean lifespan (55.7 vs. 39.4 days). (**B**) Body weight (BW) of female *Lmna*$^{-/-}$ (started with n = 12, black) and *Lmna*$^{-/-}$ *4E-BP1* (started with n = 5, green) mice were measured every-other-day started at 4 weeks of age. (**C**) Adiposity (percent body fat) was measured weekly ([fat mass/BW] x 100) from female *Lmna*$^{-/-}$ *S6K1*$^{+/+}$ (started with n = 10, black) and *Lmna*$^{-/-}$ *4E-BP1* (started with n = 5, green) mice. (**D**) Kaplan-Meier survival plot of male *Lmna*$^{-/-}$ (n = 16, black) and *Lmna*$^{-/-}$ *4E-BP1* mice (green, n = 8). Symbols represent individual mice. (**E**) BW of male *Lmna*$^{-/-}$ (started with n = 12, black) and *Lmna*$^{-/-}$ *4E-BP1* (started with n = 8, green) mice were measured every-other-day started at 4 weeks of age. (**F**) Adiposity (percent body fat) was measured weekly ([fat mass/BW] x 100) from male *Lmna*$^{-/-}$ (started with n = 8, black) and *Lmna*$^{-/-}$ *4E-BP1* (started with n = 7, green) mice.

**FIGURE 10**

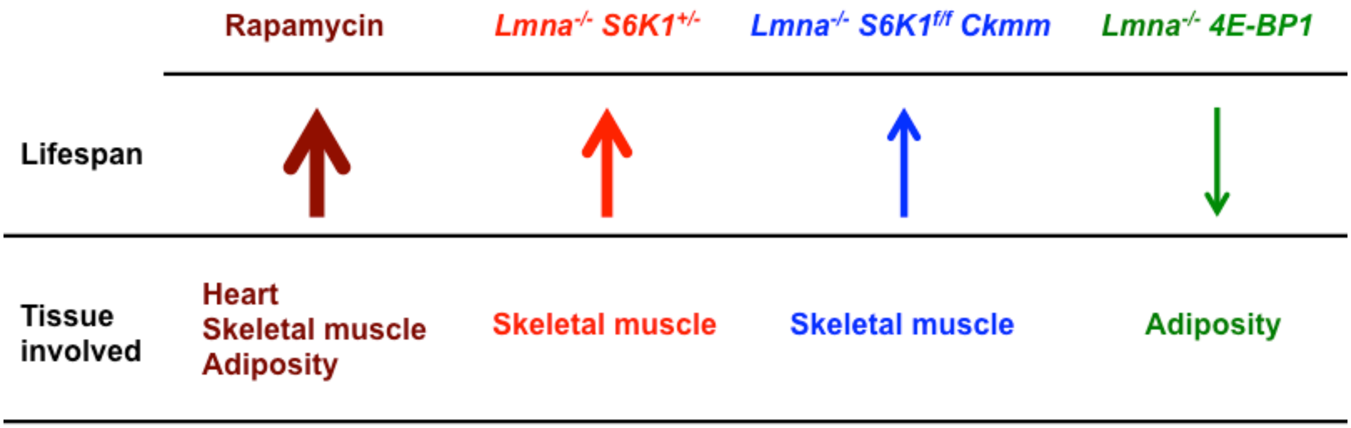


**Supplementary Figure S10. Summary of mouse models evaluated in this study.** The modulated lifespan of *Lmna*$^{-/-}$ mice by rapamycin and genetic interventions are listed as ↑: life extension and ↓: life shortening. The weight of arrows indicates the degree of lifespan modulation. Tissues that contribute to the lifespan modulation are indicated below each intervention.
